# System-Wide Associations between DNA-Methylation, Gene Expression, and Humoral Immune Response to Influenza Vaccination

**DOI:** 10.1371/journal.pone.0152034

**Published:** 2016-03-31

**Authors:** Michael T. Zimmermann, Ann L. Oberg, Diane E. Grill, Inna G. Ovsyannikova, Iana H. Haralambieva, Richard B. Kennedy, Gregory A. Poland

**Affiliations:** 1 Department of Health Science Research, Division of Biomedical Statistics and Informatics, Mayo Clinic, Rochester, Minnesota, United States of America; 2 Mayo Clinic Vaccine Research Group, Mayo Clinic, Rochester, Minnesota, United States of America; Peking University Cancer Hospital and Institute, CHINA

## Abstract

Failure to achieve a protected state after influenza vaccination is poorly understood but occurs commonly among aged populations experiencing greater immunosenescence. In order to better understand immune response in the elderly, we studied epigenetic and transcriptomic profiles and humoral immune response outcomes in 50–74 year old healthy participants. Associations between DNA methylation and gene expression reveal a system-wide regulation of immune-relevant functions, likely playing a role in regulating a participant’s propensity to respond to vaccination. Our findings show that sites of methylation regulation associated with humoral response to vaccination impact known cellular differentiation signaling and antigen presentation pathways. We performed our analysis using per-site and regionally average methylation levels, in addition to continuous or dichotomized outcome measures. The genes and molecular functions implicated by each analysis were compared, highlighting different aspects of the biologic mechanisms of immune response affected by differential methylation. Both cis-acting (within the gene or promoter) and trans-acting (enhancers and transcription factor binding sites) sites show significant associations with measures of humoral immunity. Specifically, we identified a group of CpGs that, when coordinately hypo-methylated, are associated with lower humoral immune response, and methylated with higher response. Additionally, CpGs that individually predict humoral immune responses are enriched for polycomb-group and *FOXP2* transcription factor binding sites. The most robust associations implicate differential methylation affecting gene expression levels of genes with known roles in immunity (e.g. *HLA-B* and *HLA-DQB2*) and immunosenescence. We believe our data and analysis strategy highlight new and interesting epigenetic trends affecting humoral response to vaccination against influenza; one of the most common and impactful viral pathogens.

## Introduction

Influenza infection remains a seasonal source of significant morbidity, mortality, loss of productivity, and stress. While vaccination programs have significantly reduced the morbidity and mortality of this disease and its complications, and while yearly estimates vary widely (depend on circulating strains, vaccine efficiency, etc.), on average 20,000 individuals die annually from influenza- and pneumonia-related causes in the U.S., with older persons bearing the greatest burden [1,2]. The ability of current influenza vaccines to induce protective immunity in individuals greatly determines the impact of this disease. However, studies have shown that not all subpopulations respond to influenza vaccines in the same way, to develop or maintain a protected immune state. For example, nearly 50% of the elderly have a poor influenza vaccine response and, consequently, experience increased morbidity and mortality from influenza infections [3,4].

If we can understand what causes some individuals to respond—or, alternatively, not respond—to standard vaccines, then development of alternative vaccines or immunization protocols can focus on the differential humoral, cellular, or molecular processes underlying immune response mechanisms. Numerous studies have been conducted on genotypes and gene expressions in the context of influenza vaccine response to find genetic markers that predict or explain immune response [5,6,7]. It is increasingly recognized that the immune response is a systems-level orchestration of many pathways across multiple cell and tissue types, operating on multiple timescales [8,9].

In this work, we present discovery of epigenetic markers that are associated with influenza vaccine response and, in the process, capture a more comprehensive picture of the underlying molecular mechanisms that determine response. The scope of which biologic processes are encompassed within “epigenetics” varies [10]. The covalent modification of DNA bases, specifically cytosine methylation at CG dinucleotides (CpG sites), is a commonly recognized epigenetic element that regulates gene expression, both in development (lineage determination) and in environmental response. Ongoing studies of epigenetics have been extensively reported in the literature [11,12,13,14], mostly from cancer fields [15,16,17], but also from a growing diversity of applications including imprinting [14], atherosclerosis [18,19], and aging [20,21,22,23]. Recent reviews are available from the NIH Roadmap Epigenomics Mapping Consortium online (http://www.roadmapepigenomics.org). Recently, epigenetic associations have been made between specific methylation states and response to Hepatitis B vaccine [24] and to T-cell [25,26] and macrophage [27] differentiation, indicating potential contributions to influenza vaccine response.

While many CpG sites can be readily associated with specific genes or their promoters by proximity (cis-acting) and are likely to have a direct influence on expression [28], many cannot (trans-acting) and require a broader organizing framework to facilitate their interpretation. For example, many differentially methylated regions are within enhancers and must be interpreted via transcription factor binding or other regulatory effects [29]. Gene set and pathway aggregation methods provide an organizational framework for understanding how these individual gene changes work together to orchestrate a systems-level response. Genomic annotations from previous studies were used to identify enhancers, active transcription factor binding sites, and methylation-associated regulatory regions. In addition, the co-localization of transcriptional factor binding sites indicates which transcriptional regulatory elements may be most closely related to influenza vaccine-induced immune response. Finally, we utilized network biology resources to identify enriched pathways and biologic functions associated with different methylation states, which associate with an individual’s ability to generate a protective humoral immune response. Together, these data and our analyses expand current understanding of the epigenetic regulatory landscape that is potentially active in shaping humoral immune responses to influenza vaccination.

## Materials and Methods

The subjects and methods described in this study are similar to those published for our previous studies [30,31,32,33,34,35]. High-throughput data (methylation and mRNA-Seq) used in this study are available at https://immport.niaid.nih.gov under study number SDY67.

### Study Participants

Study recruitment has been presented in our previous work [30,31,32]. Briefly, study participants (n = 158) recruited at the Mayo Clinic (ages 50–74) were generally healthy with no immune-compromising conditions. Candidates were excluded if they exhibited symptoms consistent with influenza prior to or during the study. Written, informed consent from participants was obtained at the time of enrollment and participants received one dose of the 2010–2011 licensed trivalent inactivated influenza vaccine, which contains the influenza A/California/7/2009 H1N1-like, A/Perth/16/2009 H3N2-like and B/Brisbane/60/2008-like viral strains. The Mayo Clinic Institutional Review Board granted approval for the study. Written informed consent was obtained from all study participants.

### Isolation of peripheral blood mononuclear cells (PBMCs)

The methods described below are similar to those published for our previous studies [36,37]. PBMCs were isolated from 100 mL of whole blood at each timepoint (Day 0 prior to vaccination, Day 3, Day 28). Cell processing was performed using cell preparation tubes with sodium citrate. Purified PBMCs were re-suspended at a concentration of 1×10^7^/mL in freezing medium [RPMI 1640 medium containing L-Glutamine (Invitrogen; Carlsbad, CA) supplemented with 10% dimethyl sulfoxide (DMSO; Protide Pharmaceuticals; St. Paul, MN) and 20% fetal calf serum (FCS; Hyclone, Logan, UT)], frozen overnight at −80°C, and then transferred to liquid nitrogen for storage.

### Preparation of influenza virus stock

The influenza A/California/7/2009/H1N1-like virus (Centers for Disease Control and Prevention, CDC; Atlanta, GA) was propagated in nine-day-old embryonated chicken eggs at 37°C and 82% humidity. The allantoic fluid was harvested 48 hours post-inoculation and influenza virus titers were determined by hemagglutination and the tissue culture infectious doses 50% (TCID_50_) method in MDCK cells using standard protocols [38,39,40]. To reduce variability, one viral stock was used for all assays.

### Immune Response Measurements

Influenza A/H1N1-specific humoral immunity was assessed in triplicate by HAI assay and by a memory B cell ELISPOT assay, as described previously [31,33,41]. For HAI, sera from each subject were treated with receptor-destroying enzyme, and serial dilutions were incubated with a fixed quantity of influenza virus before the addition of turkey erythrocytes to measure hemagglutination. The Day 0 intra-class correlation coefficient, measuring the correlation between replicate measurements [31], was high; 0.91.

Influenza virus-positive memory-like IgG B cells in subjects’ PBMCs were quantified in triplicate using the Mabtech ELIspot^PLUS^ kit for human IgG (Mabtech Inc.; Cincinnati, OH) according to the manufacturer’s specifications, and as previously described [31,35]. Briefly, PBMCs were thawed, plated and pretreated with R848 (1 μg/mL) and IL-2 (10 ng/mL) for 72 hours. Pre-stimulated cells were collected, counted and plated in influenza virus-coated ELISPOT plates at 2×10^5^ cells/well. Influenza A/California/7/2009/H1N1-like strain was used for the coating. Plates were incubated overnight, processed and developed according to the kit manufacturer’s specifications, and analyzed with an ImmunoSpot S6 Analyzer and ImmunoSpot v5.1 software (Cellular Technology Ltd.; Cleveland, OH). The Day 0 intra-class correlation coefficient for the B cell ELISPOT assay was 0.88.

### Illumina Human 450 Methylation BeadChip data filtering and normalization

DNA samples were randomly allocated to each plate, keeping the three time-points for each participant adjacent to each other. Beyond the laboratory quality-control metrics, data quality was assessed via probe detection rates at the 0.01 level within and across specimens, box and whisker plots, residual MVA plots, and distributions of control probes. Placental and WGA control samples were included on each plate as positive and negative controls.

Given our population of participants of northern European descent, we mapped SNPs from Refsnp [42], Hapmap [43], 1000 Genomes Project [44] that are found in Caucasian population to the CpG probe sequences on the genome. 76,670 probes with overlapping known single nucleotide polymorphism locations were removed from further analysis. We excluded 11,648 X and Y chromosome probes from our study. A further 3,085 CpG probes with ≥ 5% missing reads (across samples) were removed. As a final filtering method and in order to manage false discovery, the variance of the normalized M-values was calculated for each probe and analysis was restricted to the 101,456 probes (25%) that had the largest variability between samples and across time points.

Design I and II probe intensities were normalized separately on the log2 scale via quantile normalization. Subsequently, we applied color adjustment followed by quantile normalization of intensity values between arrays; the beta-mixture quantile normalization to align distributions between Design I and II probes [45,46]. This algorithm is similar to that proposed by Teschendorff *et al*. [45], with the order of the first two steps reversed [46]. Percent methylation values (Beta-value) were transformed to M-values by the relation, $M_{i}=log_{2}\left(\frac{\beta_{i}}{1-\beta_{i}}\right)$ for the *i*^th^ CpG site. As M-values have been shown to have more robust statistical properties [47], they were used in all statistical analyses.

### Identifying Genomic Features

#### Gene Expression

Single-end Illumina HiSeq 2000 (Illumina, San Diego, CA) mRNA sequencing was performed using the procedures outlined in our previous studies [48,49] and as presented in Ovsyannikova et. al. [50]. Briefly, Qiagen (Valencia, CA) RNeasy Plus mini Kit and the RNAprotect reagents were used to extract total RNA from 1x10^6^ PBMCs. cDNA libraries were generated using the mRNA-Seq 8 Sample Prep Kit by Illumina (San Diego, CA). Agilent 2100 Bioanalyzer (Agilent, Palo Alto, CA) was used for library validation and quantification and were loaded (5-7pM) onto individual flow cell lanes. Sequencing reads were aligned to the human genome build 37.1 using TopHat [51] (version 1.3.3) and Bowtie [52] (version 0.12.7). Gene counting was performed using HTSeq [53] (version 0.5.3p3). Within-participant differential expression was quantified over time using F-tests [46]. Differential expression was quantified across participants using gender-adjusted linear models on the log_2_ scale.

#### Gene Annotation

Methylation probes were mapped to hg19 genome coordinates using the annotation files provided by Illumina. They are associated with genes using the 2013-04-08 hg19 RefFlat file (current version available at http://hgdownload.cse.ucsc.edu/goldenPath/hg19/database/refFlat.txt.gz). We used the most upstream (towards 5’, accounting for strand) transcription start site (TSS) across transcripts defined for each gene. Promoters are defined from 500 bases into the gene to 1500 bases upstream of the gene, from the TSS and accounting for strand. Gene body regions encompass all bases from the edge of the promoter to the end of the longest transcript. Transcription Factor Binding Sites (TFBSs) and regions of open chromatin are identified from the ENCODE data tracks [54] made available through the UCSC Genome Browser [55,56]. Histone marks (H3k04me1 and H3k27ac) in lymphoblastoid (GM12878) and embryonic stem cell (H1-hESC) lines were accessed similarly. Analyses were conducted using custom scripts written in the R programming language [57] version 3.1.1, and leveraging the R packages IRanges version 2.0.1 and GenomicRanges version 1.18.4.

#### Transcription Factor (TF) Interactions

We considered trans-acting methylation sites as those that are within transcription factor binding sites (TFBSs). Their most probable functional role is to influence gene expression by altering the propensity for a TF to bind to an enhancing element. We mapped trans-acting CpGs to enhancers, open-chromatin marks, and TFBSs, generated by ENCODE and made available through UCSC. Using this TFBS annotation, we counted the number of DNA bases covered by each TF as a measure of their genome-wide prevalence. To quantify if an observed number of trans-acting CpGs in our dataset is enriched for a particular TF, we employed the null hypothesis that TFs were randomly distributed with density proportional to their genome-wide prevalence. This generated the expected ratio of CpGs overlapping a TF, given the number of CpGs under investigation. A *Χ*^2^ statistic with 1 degree of freedom (2-by-2 contingency table) was used to compare the observed and expected occurrences. TFs have different DNA binding profiles, as captured by position frequency matrices (PFMs) of aligned TF-bound sequences, which are typically summarized as binding motif logos. In order to assess the enrichment of TFs at CpG sites in greater detail, we downloaded PFMs derived from vertebrate sequences indexed by the JASPAR database [58] and computed the probability weight of CpGs (*W*_*CpG*_) occurring in each TF’s binding profile by: $W_{CpG}=\;\Sigma \;P_{i}^{C}*P_{i+1}^{G}+P_{i}^{G}*P_{i+1}^{C}$, where $P_{i}^{C}$ is the probability of observing a cytosine at the *i*^th^ position from a given TF’s PFM, and the two terms with the summation correspond to sense and antisense DNA strands.

### Statistical Methods

We associated humoral immune response outcomes with methylation probe intensities using logistic and linear regression (dichotomized and continuous endpoint, respectively). The FDA defines a sufficient vaccine response as a 4-fold increase in HAI titer (two dilution levels in our protocol) following vaccination [40]. We adjusted for each subject’s initial level (Day 0 values) in all models (change in response to vaccination). E.g. the linear model: E(Log_2_(HAI_Day28_)) = log_2_(HAI_Day0_) + w_*i*_M_*i*_. Immune outcomes were measured in triplicate. We utilized the median across replicates for both immune outcomes (HAI and B-cell ELISPOT). The models gave us a computed effect size (*w*_*i*_) and associated p-value for how informative the methylation site is for predicting immune outcome. Spearman correlation was used to assess the association between M-values and mRNA expression. We computed q-values using the approach of Storey and Tibshirani [59].

### Network Biology

We leveraged multiple network resources focused on high-confidence interactions. We combined HPRD [60], CCSB [61], the Pathway Interaction Database [62], Transcription Factor Encyclopedia [63], a directed-PPI resource [64], and a subset of STRING [65]. STRING is itself an integration of many pathway and network resources, including predicted interactions. Each interaction is provided with a confidence score. We used the subset (7.8% of interactions) of STRING where all edges have a score of at least 70%. When network resources include gene IDs (e.g., Entrez or RefSeq gene ID), we map these identifiers to current HGNC symbols. Network operations were performed using the igraph R package, version 0.7.1. Networks are visualized using Cytoscape [66] version 3.2.1.

Genesets were downloaded from MSigDB [67] v5.0, excluding those derived from genomic proximity, computational studies, and oncogenic signatures. Functional terms were also gathered from the Gene Ontology (http://geneontology.org/gene-associations/gene_association.goa_human.gz; accessed 2015-07-14) and analyzed similarly to genesets. To quantify the enrichment of genes annotated with a given functional term, we have used the standard hypergeometric test approach, where the sample (foreground) is the set of genes we have deemed “significant” by their relationship to a molecular or immunologic outcome. The background of possible genes for each test is the intersection of genes indexed by the resource (e.g., MSigDB and GO) and those assayed by RNA-Seq within our dataset.

## Results

### Data Quality

One sample had systematic low performance and failed to produce usable data from the array and was excluded from further analysis. There were 158 subjects used in the analysis; 61% were female, with an age range of 59–73 years. All but two subjects were Caucasian. Overall, methylation changed little over time (not shown). Day 0 methylation levels are thus used, unless stated otherwise.

### Methylation-Expression Associations

**Fig 1:**
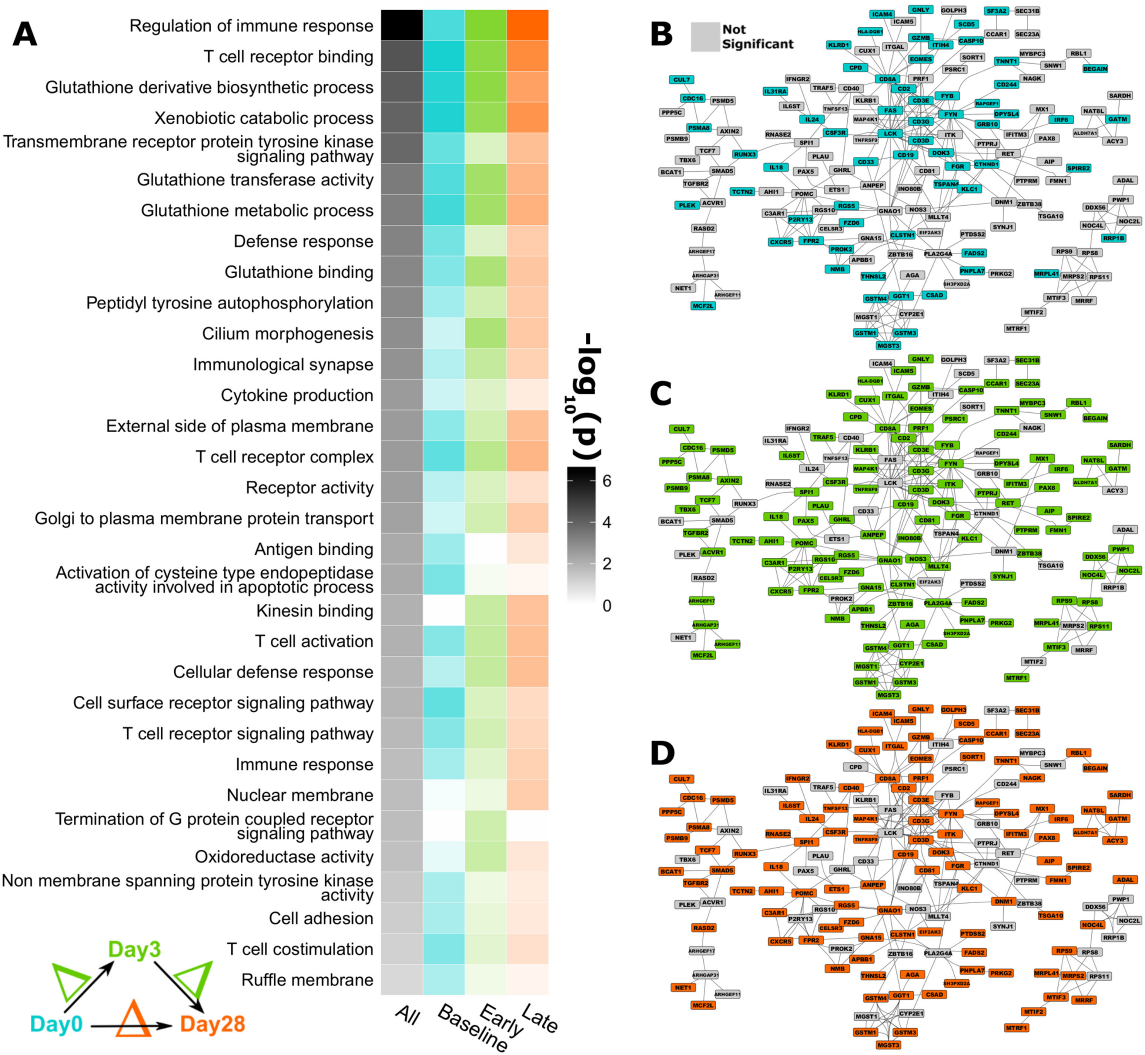
Genes whose expression is highly correlated with cis-acting CpGs show functional enrichment. **A)** Genes with significant association (p < 1E^-4^) indicate 32 GO terms enriched at the p < 0.01 level and annotating at least 3 genes, across time points. Color intensity is used to signify statistical significance. Genes are mapped to network biology resources (see Methods) and the associations at **B)** baseline, **C)** during early and **D)** late time periods shown, represented in the same location in all panels; (for brevity, only genes within the largest connected components are shown). We color genes in the network that have a significant association at each time period (baseline teal, early green, late orange). The network layout is manually adjusted and edges bundled to improve legibility. See the online version for sufficient resolution to view gene names.

We considered the correlation between methylation loci and cis-gene expression independent of immune outcome. Table 1 lists the top 20 cis-associations between Day 0 methylation levels and Day 0 gene expression measured by RNA-Seq, for the average methylation level across each genomic region (gene promoter or body). See S6 Table for the full list, and for correlations with gene expression at later time points. The system-wide summary of differential (through time) cis-acting methylation is shown in Fig 1, using the genes whose expression is correlated with a cis-acting CpG with p < 1E^-4^; 716 CpG sites in total, with the 161 genes sharing network connections displayed in Fig 1. We show the same network configuration with color indicating three different temporal states: Baseline (Day 0, pre-vaccination); Early (Day 3); and Late (Day 28) immune response after vaccination. While Day 28 is approximately a peak of adaptive response [68,69], we refer to it as “late” in this study for simplicity. The genes meeting the aforementioned significance threshold were carried on to GO term [70] enrichment, which identifies participation in similar biologic functions. Further, this group of genes with significant methylation-expression associations are depleted (compared to randomly selecting 716 genes) for network interactions (p = 0.028), potentially indicating their diverse functional roles. Interestingly, there are many genes in common between the three time states; 28.8% are significant in all three, and 61.7% in at least two (S1 Fig).

**Table 1:** Genes for which cis-acting methylation sites are highly correlated with expression.

Gene Promoter^‡^				Gene Body			
	r^†^	p-value	q-value		r	p-value	q-value
LOC654433	-0.89	6.17E-54	5.60E-50	PAX8	-0.77	1.77E-31	1.30E-27
DDX43	-0.82	1.35E-39	6.13E-36	LOC654433	0.75	1.53E-29	5.64E-26
PM20D1	-0.78	1.19E-33	3.60E-30	TMEM8A	-0.74	8.97E-29	2.21E-25
ZNF714	-0.76	6.80E-31	1.54E-27	DDX43	-0.73	3.15E-27	5.81E-24
IRF6	-0.76	1.19E-30	2.15E-27	GLB1L	-0.72	9.64E-27	1.42E-23
NLRP2	-0.75	1.51E-29	2.28E-26	PRSS21	-0.71	7.43E-26	9.14E-23
LOC391322	-0.73	1.42E-27	1.83E-24	MDGA1	0.69	2.85E-23	3.00E-20
ZFP57	-0.69	2.03E-23	2.30E-20	LOC253039	-0.68	1.37E-22	1.27E-19
NLRP2	-0.68	8.85E-23	8.91E-20	MRPL21	-0.68	3.00E-22	2.46E-19
HLA-DQB1	-0.68	1.09E-22	9.92E-20	PNMAL2	0.67	2.12E-21	1.47E-18
AMDHD1	-0.68	2.04E-22	1.68E-19	PPP5C	-0.66	2.19E-21	1.47E-18
C8orf31	-0.66	2.37E-21	1.79E-18	NLRP2	-0.66	3.98E-21	2.45E-18
LCLAT1	0.66	4.25E-21	2.96E-18	FADS2	-0.66	5.02E-21	2.85E-18
PRSS21	-0.66	4.64E-21	3.00E-18	SRXN1	-0.64	1.07E-19	5.63E-17
DNAJC15	-0.65	7.00E-20	4.23E-17	QDPR	-0.64	2.06E-19	1.01E-16
TNNT1	0.64	1.72E-19	9.75E-17	AGAP4	0.64	2.24E-19	1.03E-16
GTSF1	-0.62	7.58E-18	4.04E-15	VARS2	-0.64	2.89E-19	1.25E-16
IL32	-0.61	2.71E-17	1.36E-14	PRKG2	-0.63	4.91E-19	2.01E-16
NAPRT1	-0.60	6.65E-17	3.17E-14	AMDHD1	0.63	7.76E-19	3.01E-16
HOXC4	0.60	9.20E-17	4.17E-14	POMC	-0.63	1.29E-18	4.77E-16

^†^r is the Spearman’s correlation coefficient.

^‡^The regionally averaged methylation levels are used; see Methods.

For Day 0 methylation and expression data, 1,127 individual promoter CpGs are correlated with the cis-gene’s expression at the q ≤ 0.01 significance level. This is 2.5% of the 40,483 promoter CpGs, and 4.2% of the 26,817 promoter CpGs with expression of the cis-gene in our dataset (robustly assayed and with non-trivial variance across participants). Using methylation levels averaged across the gene’s promoter, 346 out of 11,517 promoters (3.0%) have significant associations at same significance level, 174 (50%) of which also have at least one individual CpG with a significant association with cis-gene expression.

### Humoral Immune Response Associations

**Fig 2:**
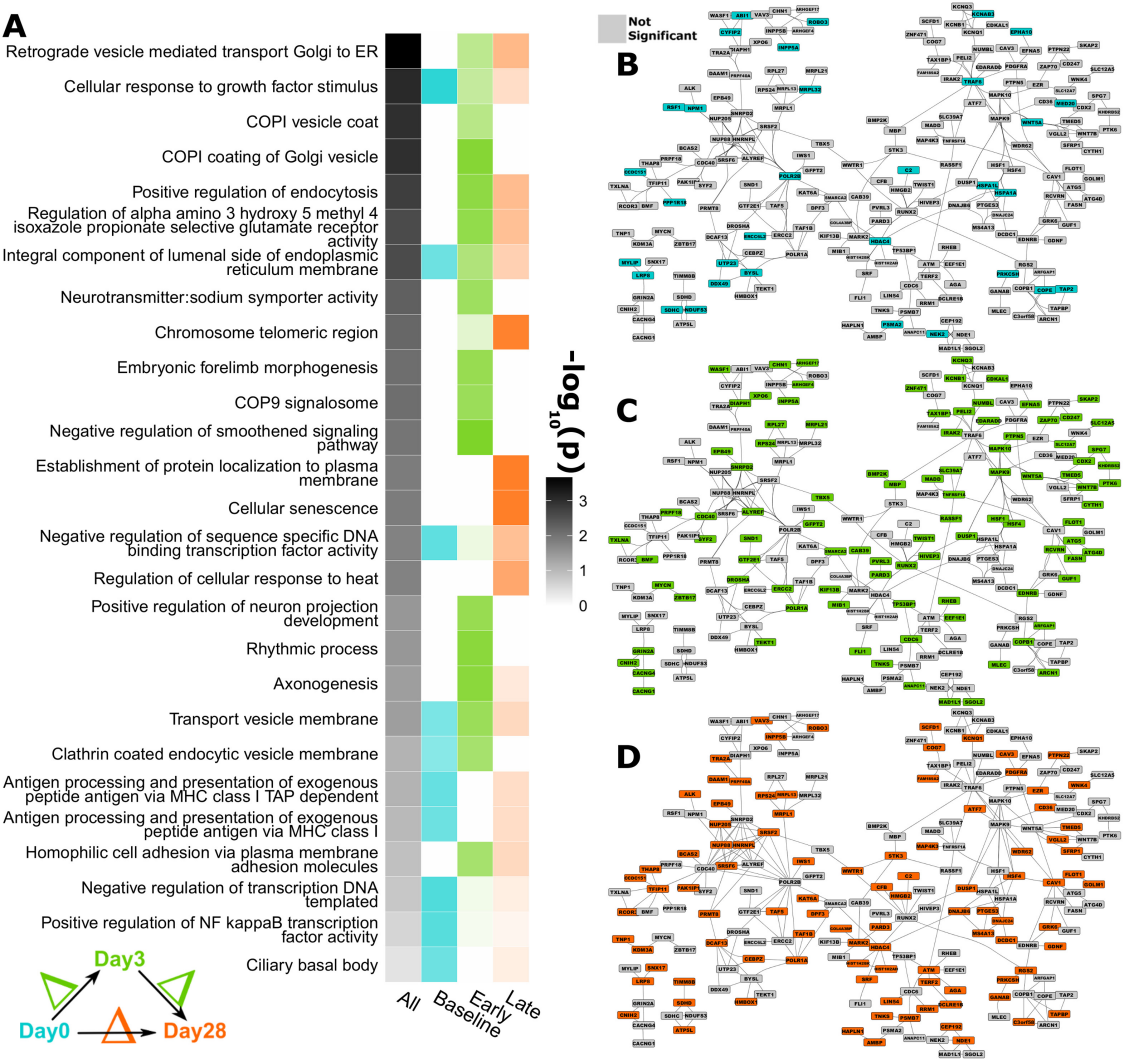
Methylation-HAI network based on linear regression models. Day0 methylation levels of cis-acting CpGs and the change in HAI titer between Day28 and Day0 are used to generate linear regression models. Coloring and display is as in Fig 1.

We analyzed the association between baseline methylation and HAI at multiple timepoints using linear regression models, showing the top results in Table 2 and the full list in S7 Table. Cis-acting CpGs showed temporal patterns of biologic function enrichment with new and differentially activated biologic processes observed through Day 3 and Day 28 (Fig 2). Few genes (8.2%) are recapitulated at a second timepoint (S1 Fig). The strongest statistical associations are plotted in S5 Fig, and are for the HLA genes *HLA-DQB2* (p = 9.57E^-6^; q = 0.38) and *HLA-B* (p = 1.08E^-5^; q = 0.38).

**Table 2:** Influenza HAI linear models utilizing Day 0 methylation.

CpG (M_*i*_)	ΔHAI for Δ*M*_*i* Q3 to Q1_^†^	p-value	q-value	GenomicRegion	Gene
cg15321244	-0.81	9.57E-6	0.38	GeneBody	HLA-DQB2
cg23923934	-0.58	1.08E-5	0.38	GeneBody	HLA-B
cg02914652	0.63	1.41E-5	0.38	Open, TF	-
cg10544627	-0.53	1.64E-5	0.38	Open, No TF	-
cg00016156	-0.44	2.71E-5	0.42	Promoter	MIR3912;NPM1
cg04483460	-0.53	2.73E-5	0.42	GeneBody	LRP8
cg13022911	-0.42	3.43E-5	0.43	Promoter	LOC401980;TMEM183A; TMEM183B
cg18001427	-0.59	3.71E-5	0.43	Promoter	RWDD2B
cg12625454	-0.51	4.29E-5	0.43	GeneBody	PTPRN2
cg06824297	-0.38	4.67E-5	0.43	Promoter	RWDD2B
cg14311250	-0.49	5.69E-5	0.48	Open, No TF	-
cg11705439	-0.45	7.96E-5	0.52	Promoter	CCDC151;PRKCSH
cg19333739	-0.50	8.04E-5	0.52	-	-
cg20803547	-0.50	8.36E-5	0.52	Promoter	IL12RB2
cg20404355	-0.50	8.41E-5	0.52	-	-
cg11194925	0.60	9.15E-5	0.53	GeneBody	PAX9
cg01109337	-0.49	1.04E-4	0.53	GeneBody	TEX14
cg10016610	0.17	1.07E-4	0.53	GeneBody	ROBO3
cg15249796	-0.44	1.25E-4	0.53	GeneBody	TEX14
cg12390946	-0.50	1.29E-4	0.53	Promoter	INPP5A

All models were adjusted by baseline HAI values.

^†^ We express the effect size of linear regression models in terms of the change in HAI titer predicted from the model for participants at the Q3 (75^th^) percentile of methylation M-value relative to Q1 (25^th^).

Starting from candidate genes that show within-person changes over time, we identified the associated changes in cis-acting CpG methylation. A concise list of genes exhibits both associations: *TAF5*, *PAX6*, *EPHB1*, *CCDC23*, *CTR9*, and *CWC22* exhibit both cis-CpG association (p < 0.01) and HAI association; *ZNF628*, *TMF1*, *CKAP5*, *TNFAIP1*, and *RUNDC1* exhibit both cis-CpG association (p < 0.01) and B-cell ELISPOT association.

Next, we averaged methylation levels across shared genomic regions (e.g. multiple cis-acting CpGs within a gene’s promoter) and quantified their association with humoral immune outcomes (strongest associations are shown in S3 Table). Regions with greater heterogeneity between probes (see S2 Fig for examples) are more likely to show differences compared to the per-CpG analysis when averaged, than more uniform regions. By comparing the genes identified by each method (logistic and linear regressions, per probe and regionally averaged), we observed greater concordance among per-CpG analyses than among regionally averaged analyses (S3 Fig). However, each HAI-centric method showed associations with different genes.

We next considered trans-acting CpGs as those that are not easily associated with a specific gene via proximity in the genome, and annotated them with using TFBSs and histone marks. We summarize the overlapping TFs across trans-acting CpGs in S1 Table. The overall occurrence pattern of TFBSs is similar to that of the whole genome, with CpGs most often occurring at positions important for nucleosome spacing (*CTCF*) and global growth and development (*MYC*/*MAX*). Typically, multiple TFs overlap each locus, likely indicating combinatorial regulation at each site. Methylation of these TFs’ binding sites may have important impacts on modulating immune cell growth and differentiation rates and influencing HAI via this mechanism.

CpGs overlapping accessible TFBSs shows the potential for a regulatory role, but further association with established enhancer marks via histone H3K4 mono-methylation (H3K4me1) and H3K27 acetylation (H3K27Ac) provides greater regulatory evidence. Considering regions of the genome outside of gene promoters and gene bodies, we calculated the fraction of bases associated with one of these histone marks to be 18%. The analogous background rate within the filtered CpGs used in our analysis (n = 101,456) is 74%. H3K4me1 peaks overlap 42 of our 154 HAI-associated trans-acting CpGs, while H3K27Ac overlaps 86. Together, the two histone marks overlap 95 (62%) CpGs; this is an overall enrichment (one-sided exact binomial test p-value < 1x10-16), but slightly depleted compared to expectation for the chip in general (p = 5.2x10-4). Similarly, of the 135 trans-acting CpGs associating with B-cell ELISPOT outcomes across timepoints, 35 overlap H3K4me1 and 68 overlap H3K27Ac, with 81 (60%) in the union of the two.

**Fig 3:**
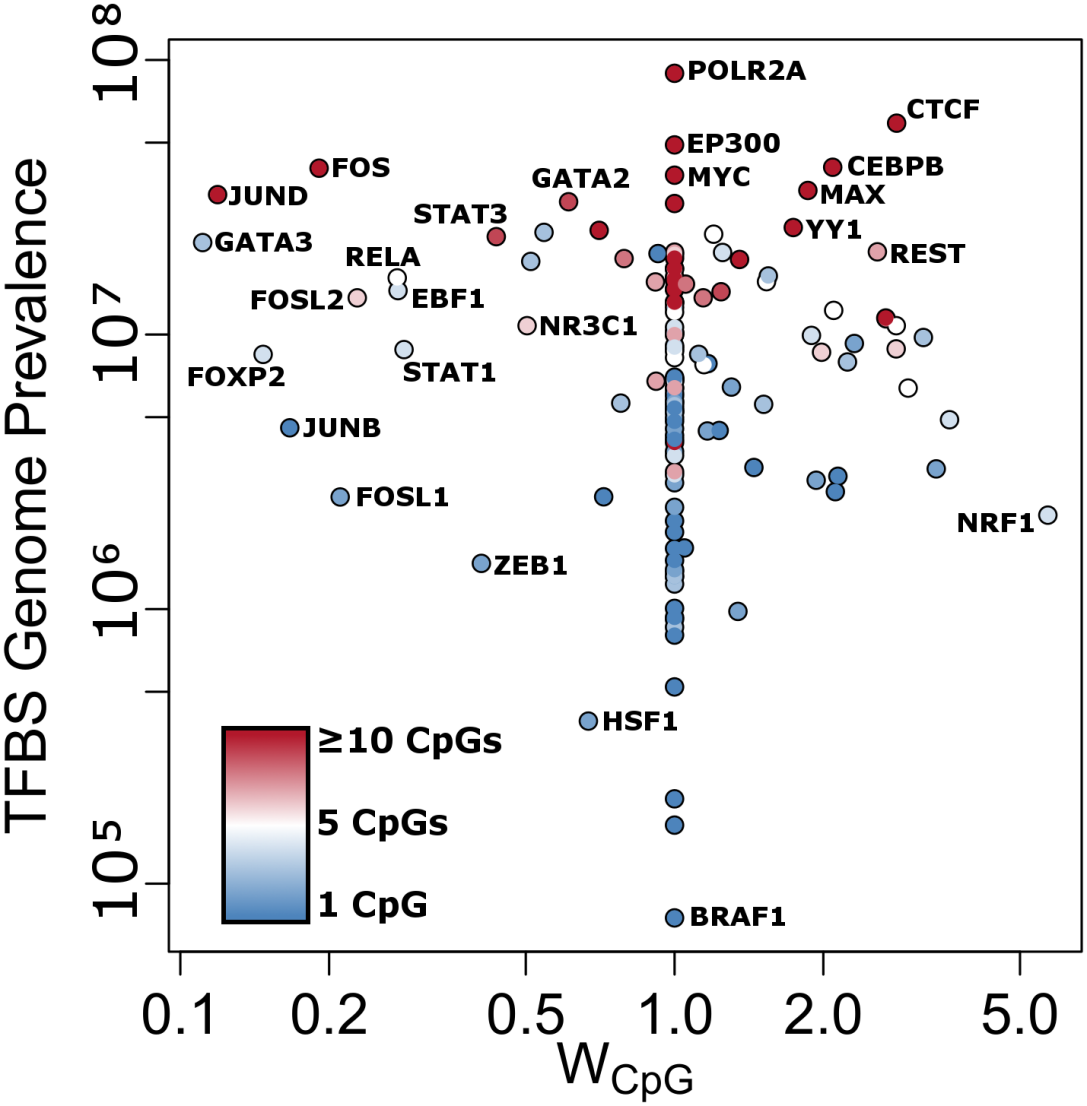
Trans-acting CpGs whose baseline levels correlate with HAI titer and that overlap TFBSs are annotated by the TF’s genome-wide prevalence and the probability for the TF’s binding motif to contain CpGs. The probability weight, W_CpG_, corresponds to the probability that a TFBS motif will contain the indicated number of CpGs. If a TF is not indexed by JASPAR, it is given a W_CpG_ value of 1.

We also considered the level to which we’d expect to observe TFBSs compared to randomly selected loci. We quantified the enrichment of each TF among the 154 TFBS-overlapping CpGs that also had an HAI titer-association, normalized by each TF’s prevalence across the genome. For CpGs significantly associated with HAI, we identified the transcription factor *EP300* (10 CpGs; p = 8.37E^-2^) as under-represented and *EZH2* (21 CpGs; p = 4.79E^-3^), *CTBP2* (11 CpGs; p = 2.34E^-2^), and *SUZ12* (7 CpGs; p = 7.33E^-2^) as over-represented (see S1 Table). Finally, we weighted TFs by their probability of binding a DNA sequence containing a CpG site (Fig 3). In addition to being prevalent, TFs that are more likely to bind CpG-rich sequences include *CTCF*, *CEBPB*, *MAX*, and *YY1*. However, TFs that are unlikely to bind CpG-containing sequences, but which overlap multiple HAI-associated CpG loci, include *JUND*, *JUNB*, *FOS*, *FOSL2*, *FOSL1*, *STAT3*, *STAT1*, *GATA2*, *GATA3*, *RELA*, and *FOXP2*.

We applied the same analysis strategy for B-cell ELISPOT outcomes. The most significant per-CpG results are shown in S4 Table. Among these top associations are *PIEZO1* (p = 1.34E^-6^; q = 0.14), *KRT7* (p = 3.71E^-6^; q = 0.15), *HDAC4* (p = 4.44E-5; q = 0.64) and intergenic CpGs within regions of open chromatin with known TFBSs. GO term enrichment and the implicated sub-networks are shown in S4 Fig. The strongest regionally averaged methylation associations with B-cell ELISPOT level is *TTC5* (p = 2.37E^-6^; q = 4.25E^-2^) promoter methylation. Finally, we applied the same methodology to quantify the enrichment of 135 TF among TFBS-overlapping CpGs that also have a B-cell ELISPOT association (see S1 Table). We identified *POLR2A* (20 CpGs; p = 8.96E^-2^) as under-represented and *YY1* (22 CpGs; p = 2.20E^-2^), and *FOXP2* (10 CpGs; p = 8.81E^-2^) as over-represented. Considering the likelihood of TFs to bind CpG-containing sequences, TFs very similar to the above for HAI are highlighted, but with increased representation for *GATA3*, *RELA*, and *FOXP2*.

### Integrating Expression and Outcome Associations

**Fig 4:**
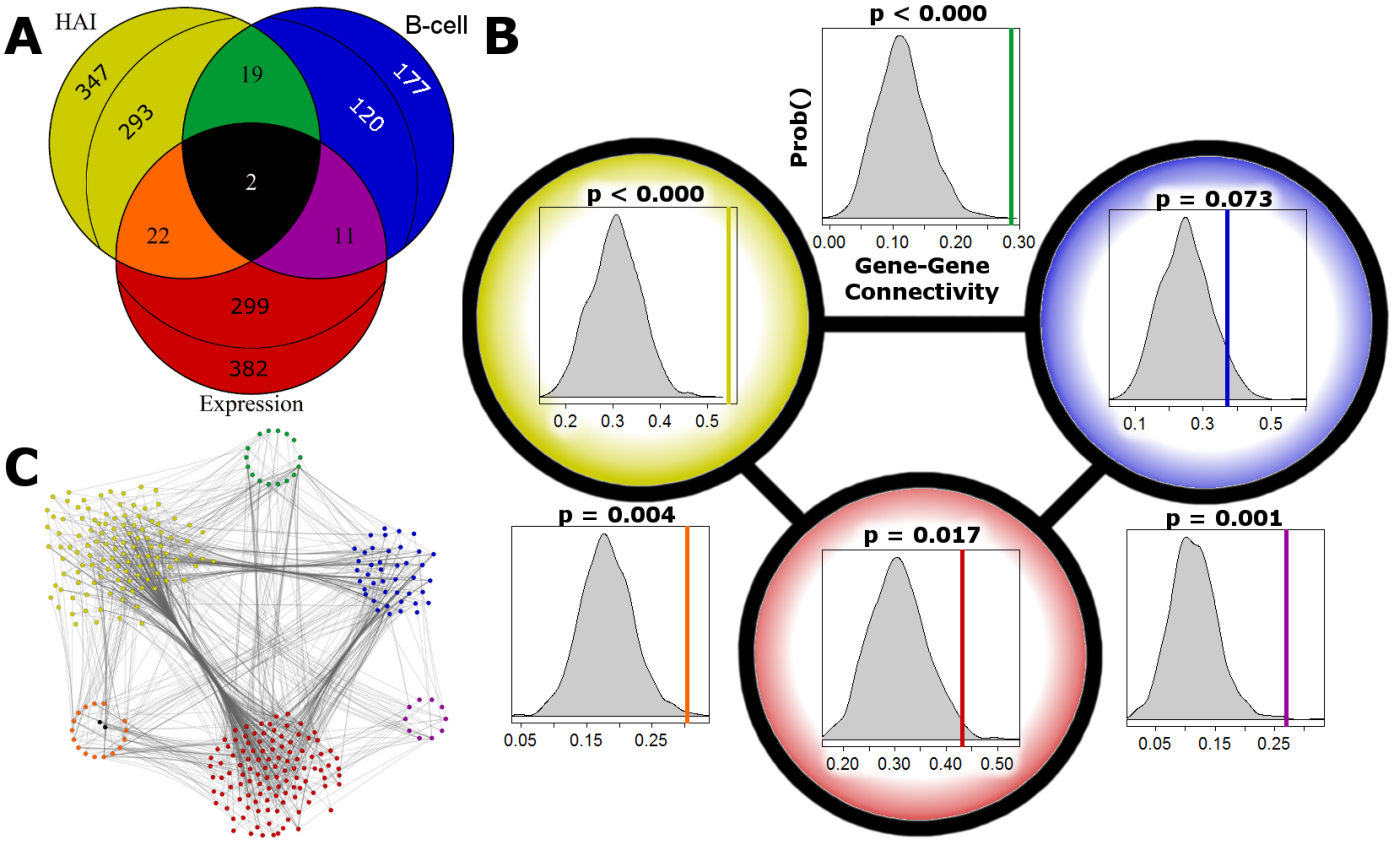
Interrelationships between genes associating with each humoral immune outcome. **A)** A Venn diagram summarizes the number of genes associated with each outcome across time points. A “core” set of 54 genes are identified using at least two outcomes. We divide genes only associated with one outcome into two groups; those that share network links with genes in the core (inner number), and those that do not (outer number). **B)** The fraction of genes with direct links to each other (gene-gene connectivity), within and between each outcome-specific set, is compared to observations from random gene sets of the same size. The six comparisons between HAI, B-cell ELISPOT, and gene expression are shown in the same relative position as the Venn diagram with a colored vertical bar indicating the gene-gene connectivity observed in our study and the distribution of connectivity from randomly generated genesets in gray. **C)** A visualization of the network is shown using the subset of links with greatest confidence and laid out similarly to the other panels. The extent of gene-gene connectivity is apparent from the number of genes (represented by colored circles) with known direct interactions (gray lines crossing between groups).

We have identified associations between DNA methylation, gene expression, and humoral immune outcomes. Next, we compared and contrasted these analyses (Fig 4). In order to integrate across cis-acting associations using both individual CpG and promoter-averaged analyses, all results are summarized to the gene level. First, we identified cis-acting methylation with associations in at least two analyses and list them in Table 3. Two genes show association in all three analyses: ADARB2, an inhibitor of adenosine deaminase activity (RNA editing), and SPEG, a kinase with known function in myocyte development. Second, subsets of CpGs that predict late response also show concordance with low baseline activation (S6 Fig). Participants with low methylation within a particular cluster of CpGs show lower baseline log_2_ B-cell ELISPOT values (2.7±1.8) than participants with high methylation of levels of the same CpGs (3.4±1.4). Third, known interactions between these genes from network biology resources are gathered and the level of interaction between them tested for statistical significance. Many of the genes identified in one analysis have direct interactions (protein-protein interaction or one link apart in known pathways) with genes identified in the other analyses. For example, there are 640 genes identified in the analyses focused on B-cell ELISPOT or gene expression. Similar ratios are seen starting from B-cell ELISPOT associated genes (40%) and expression- level associated genes (44%). Beyond this highly connected core, we tested the level of interconnectivity (fraction of genes sharing links) between the genes showing association with only one outcome. Comparing our observed levels of gene-gene interconnectivity to distributions of randomly selected genes of the same sizes, the observed levels are high (p < 0.005) for all three pairwise comparisons (Fig 4).

**Table 3:** Genes showing associations in multiple analyses.

Methods	N	Gene Symbols
Methylation^†^-Expression^‡^ & Methylation-HAI	22	AATK, AGA, ARHGEF17, C16orf55, DPY19L2P2, EBF4, FAM24B, FAM24B-CUZD1, FAT4, HCP5, IGHMBP2, MRPL21, PCDHGA4, PCDHGB2, PPFIBP2, PTCD3, RASSF1, RDH13, RWDD2B, SLC12A7, WWTR1, ZNF418
Methylation-Expression & Methylation-B-cell ELISPOT	11	APOLD1, ARHGEF10, CORO2B, IL6ST, LACC1, MCF2L, NAPRT1, PLEKHN1, SDR42E1, SPATC1, TNFRSF9
Methylation-HAI & Methylation-B-cell ELISPOT	19	ANGEL2, C2, CDC40, FLOT1, HCN2, HDAC4, JPH4, METTL22, NXN, PTPRN2, RCOR3, SLC6A19, SLFN13, SORCS3, TMEM132C, TNRC18, TTC40, UNC13A, ZBTB12
All 3	2	ADARB2, SPEG

^†^ Genes are represented by average probe intensities for cis-acting (promoter or body) CpGs.

^‡^ Genes are represented by their normalized RNA-Seq expression levels.

## Discussion

In order to better understand immune response to influenza vaccine in the elderly, we investigated the changes in and associations between genome-wide DNA methylation, gene expression, and humoral immune response outcomes in a cohort of 158 50–74 year old individuals. We did not identify large changes in methylation patterns after vaccination. Given the important role of epigenetic modification during T cell differentiation [26] and response to infection [71], this finding was unexpected. It is likely that parenteral administration of a split-virion vaccine induces a much smaller epigenetic response that a live viral infection. Another factor to consider is the use of PBMC samples in our study instead of purified cell subsets, which may have diluted changes in cell subset-specific DNA methylation patterns.

In contrast, strong cis-CpG associations with gene expression were revealed. The impact of methylation on humoral immunity is complex and highly dependent upon the immune outcome and the CpG handling–the measurement (HAI versus B-cell ELISPOT), resolution (continuous or dichotomized), and data handling (per-CpG versus regionally-averaged). These differences in prioritization potentially indicate the breadth of regulatory marks involved in a system-wide response.

First, we were interested in which sites’ methylation levels have a strong association with gene expression. Methylation levels in gene promoters and gene bodies have a direct influence on whether the gene will be expressed. We have referred to such sites as cis-acting and present their enriched effects (GO terms) in Fig 1. Because of other regulatory interactions, methylation is not necessarily deterministic for expression levels, but can have a significant effect on the likelihood that a gene will be expressed. For example, we find a strong negative correlation (r = -0.61) between *IL32* promoter methylation and *IL32* gene expression. *IL32* is a pro-inflammatory cytokine that plays an important role in inducing INF-α production. When differences in *IL32* activity are seen between individuals, a strong component of this difference is likely due to differential methylation.

In Table 1 we list the genes with highest correlation between average promoter (and gene body) methylation and expression of the same gene. Functional term enrichment is an efficient way to summarize themes common to groups of genes. Without focusing the analysis on immune outcome, the most enriched GO terms among these genes include glutathione regulation, T-cell receptor complexes, antigen and cytokine production, and other immune-related functions (Fig 1). The observed baseline (pre-vaccination) enrichment of immune-related genes is likely due to the standard expression profile of PBMCs (immune cell-enriched expression). It is the change in activation over time that is most directly a result of vaccination. Due to the high fraction of genes with shared methylation-expression associations between timepoints, the statistically enriched GO terms are often enriched across timepoints. Glutathione is a potent anti-oxidant. Differential regulation of glutathione could impact overall stability or robustness of the immune cell population. We find more associations for individual CpGs than for methylation averaged across the promoter. This is likely to have contributions from multiple effects including the combinatorial nature of gene promoters [72]. Such trends, while not necessarily directly associated with immune outcomes, are likely to have an indirect impact via age-associated immune-senescence, or via differential activation of immune-related pathways.

Focusing the analysis on cis-acting CpGs (within a gene promoter or body) whose methylation level is associated with HAI, more granular terms are prioritized. For example, vesicle trafficking, antigen processing, TLR3 signaling, and multiple cellular differentiation pathways are significantly enriched (Fig 2). Within vesicle trafficking, COPI complex genes have been shown to play a role in the phagocytic process in other pathogenic contexts [73,74], making a plausible mechanism for their strong association with HAI in our study; determining the extent of immune reaction to vaccination. Unsupervised clustering of humoral immune outcome-associated CpGs identified a group of subjects with low relative methylation across a cluster of CpGs and lower B-cell ELISPOT values (S5 Fig).

We also analyzed the association of HAI using a dichotomized endpoint according to FDA guidelines (responder or non/low responder), using logistic regression models. The top CpGs are shown in S2 Table. Due to the large number of statistical comparisons, all q-values were large. However, some of the marginal results may be of some interest due to their known immunologic role. For example, methylation levels of a CpG within the gene body of *HLA-B* were strongly associated with HAI response. Specifically, participants in the 75^th^ percentile of methylation were at 1/3 the odds of being a responder as their contemporaries in the 25^th^ percentile. A second example is *HCP5* (HLA-complex 5 lincRNA) for which an opposite trend is observed.

We found a relatively small number of overlapping genes among our HAI-centric analyses (S3 Fig). The genes overlapping across methods and at multiple time points include *HLA-DQB2*, *RWDD2B*, *PTPRN2*, *DNAH2*, *HCP5*, *FAM24B*, and *LOC399815*. *HLA-DQB2* is a class-II antigen-presentation gene; a gene vital to immune response. *PTPRN2* has been shown to (de)phosphorylate phosphoinositols leading to an insulin regulatory role [75]. Further genes associated in at least two time points include the class-I antigen presentation gene, *HLA-B*, histone deacetylase *HDAC4*, interleukin receptor *IL12RB2*, and the transcription factors *PAX7* and *PAX9*.

There are multiple contributing factors to the observed extent of overlapping genes: First, we have assumed in our linear models that the change in HAI defines vaccine response. However, in the logistic regression models, a subject may meet the approved definition of “protected,” but lack any change over time (and vice versa) [32]. Second, in averaging across genomic regions (e.g., gene promoters), real biologic signals may be lost, especially for transcript-specific effects. When multiple CpGs occur within a region, it may be that one (or a few) carry a greater influence, for a given condition (e.g., vaccination). Alternatively, there could be one CpG that has no influence. In both cases, averaging may dilute the real signal. For this reason, some genes have significant associations in our per-CpG analysis, which no longer have a significant association when averaged, and vice versa.

Trans-acting CpGs overlapping TFBSs were tested for commonly regulated TFs (S1 Table). CpGs that have significant associations with HAI show enrichment for *EZH2* and *SUZ12* binding sites. Both of these TFs are members of Polycomb Recessive Complex 2. Considering B-cell ELISPOT outcomes, a similar analysis highlights *YY1* as being enriched. *YY1* assists Polycomb Complexes [76], and is itself a broadly acting TF. Together, both humoral immune outcomes point to genomic enhancers differentially methylated and potentially affecting Polycomb Complex regulation. Further, multiple CpGs associating with B-cell ELISPOT regulate *FOXP2* binding sites. *FOXP2* has a known role in neurodevelopment [77], and its specific role in immune development or response may warrant further investigation. TFBS motifs are consensus-based models used to summarize common sequence features. Recent chromatin immune-precipitation (ChIP) and ChIP-exo analyses demonstrated that not all sequences found bound to a TF contain the canonical motif [78,79,80]. We have annotated TFs by their likelihood of binding to CpG-containing sequence. This annotation further highlights *FOXP2* (Fig 3). Multiple TFs with known inflammatory and immune response functions are also highlighted by their low probability of binding CpG sequences, but with high representation at CpGs whose levels associate with HAI and B-cell ELISPOT outcomes.

While the individual genes prioritized by the previously discussed analyses differ, the biologic functions that they regulate are inter-related. The known relationships between these genes (network connections) highlight their shared functional roles. Since “links” between genes have been selected primarily from previously reported protein-protein interactions and pathway relationships, we assume high connectivity indicates functional relationships. Genes with cis-acting CpGs associated with HAI, B-cell ELISPOT, or expression of the same gene, all share a high degree of network interactions compared to the same number of randomly selected genes (Fig 4). While, a portion of the genes showing associations could be spurious, the extent of interaction among them, and biologic plausibility, point to common functional roles identified by our different analysis strategies.

The methylation data in our study was generated using the Illumina Infinium Human Methyl450 BeadChip [81,82], and whole PBMCs. PBMCs contain approximately 15% B-cells, 15% NK cells, and 70% CD3+ T-cells, each of which can be further subdivided. For example, CD4+ and CD8+ T-cells can be separated or further subdivided into Th1, Th2, and Th17 regulatory T cells. While some of these subpopulations will respond to the vaccine antigen congruently, there will be stratifications, such as naive and memory cells, that may respond differently [83,84,85]. Identifying markers at these finer fractions is much more complicated and detracts from the goal of an ideal biomarker that can be detected with minimal or no sample manipulation.

Because CpG methylation rates will vary between cells and cell lineages, when measured as a heterogeneous population, it will reflect the relative fraction (0% to 100%) of cells that are methylated on one or both diploid copies of each site. When the epigenetic state of a given regulatory site influences one cell type specifically, we would expect a small overall effect size when measured in PBMCs. This makes even small absolute changes in methylation at the extreme ends (e.g., moving from 5% to 1% methylated at a given site) of potential functional significance. By comparing to reference datasets derived from individual cell subsets, signals can be associated with the cell subsets most likely to be active; a process referred to as deconvolution [86,87].

The data presented here could be expanded in future work. For example, recent studies have shown that the maximal B-cell response is likely to be observed 5–10 days after vaccination [88]. In this study, we observed significant changes in B-cell ELISPOT levels by 3 days post-vaccination and present methylation data associated with these changes. Unfortunately, we do not have biospecimens from 5–10 days post-vaccination to compare to our earlier timepoint. Further, we have identified different associations when analyzing out data on a per-CpG basis, or averaged across genomic regions (e.g. gene promoters). Assigning the relative strength of each CpG within each genomic region may be possible given sufficient samples and the genomic relationships available through ENCODE. Expansion of this work to larger datasets and integration with further data types (e.g. miRNA, or immunosenescence markers across the Human Immunophenotype Consortium; http://www.immuneprofiling.org) will give a more complete picture of the systems-level response to vaccination.

In summary, we identify a broad list of CpG sites showing associations with gene expression and vaccine-induced humoral immune outcomes. While few humoral immune outcome associations reach false-discovery-corrected significance, prior knowledge of immune-related roles for the genes indicates plausible functional associations that will inform a systems biology view of vaccine response. Network resources indicate many interactions among these genes, providing further evidence of functional associations. We believe our data indicate compelling epigenetic trends that play a role in determining humoral response to vaccination against influenza.

## Supporting Information

S1 Fig Across-time methylation associations with expression are more stable than with humoral immune outcomes. (DOCX)

S2 Fig Examples of gene promoters exhibiting different levels of consistency between CpGs sites. (DOCX)

S3 Fig Comparison of the number of genes shared by HAI association methods. (DOCX)

S4 Fig Examples of regulatory network interactions implicated by CpG sites whose methylation levels correlate with B-cell ELISPOT response. (DOCX)

S5 Fig The most significant associations from linear models of methylation versus HAI titer are for A) HLA-DQB2 and B) HLA-B. (DOCX)

S6 Fig Unsupervised clustering reveals moderate concordance between baseline (pre-vaccination) B-cell ELISPOT (BCE) levels and CpG methylation. (DOCX)

S1 Table Most frequent TFs binding to the most informative CpGs. (DOCX)

S2 Table Influenza HAI logistic models utilizing Day 0 methylation. (DOCX)

S3 Table Spearman correlation between the average baseline methylation level (across probes) of gene promoters and gene bodies with influenza HAI. (DOCX)

S4 Table B-cell ELISPOT response linear regression models. (DOCX)

S5 Table Spearman correlation between the average methylation level (across probes) of gene promoters and gene bodies with the change in B-cell ELISPOT from Day 0 to Day 28. (DOCX)

S6 Table Correlations between baseline methylation levels and gene expression, across time points, for all genes with analyzed cis-acting CpGs. (XLSX)

S7 Table Linear model results between HAI and methylation levels at multiple time points, for cis-acting CpGs. (XLSX)
