# Supplementary material for: System-Wide Associations between DNA-Methylation, Gene Expression, and Humoral Immune Response to Influenza Vaccination
Source: PLoS One. 2016 Mar 31;11(3):e0152034. doi: 10.1371/journal.pone.0152034 (PMC4816338; doi:10.1371/journal.pone.0152034)
Supplement: S1 Fig — (DOCX) [file pone.0152034.s001.docx]

Expression HAI B-Cell ELISPOT


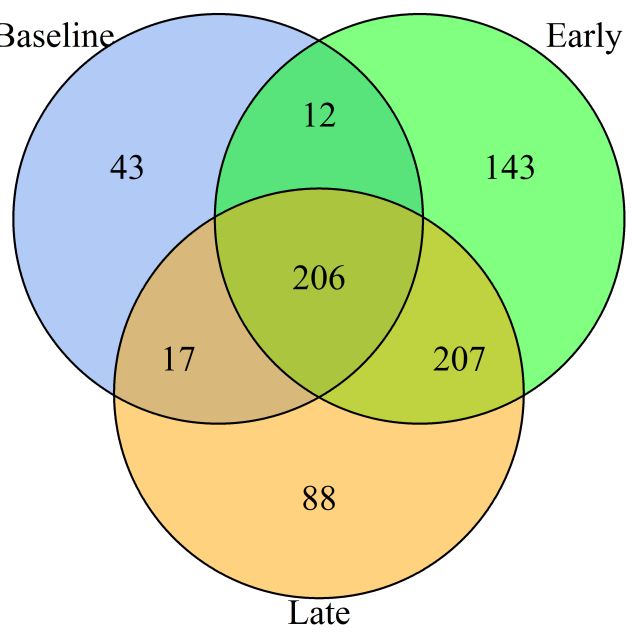

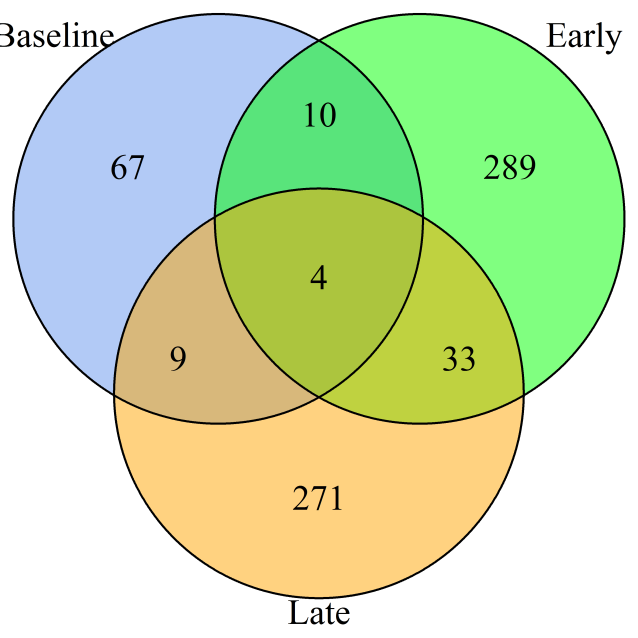

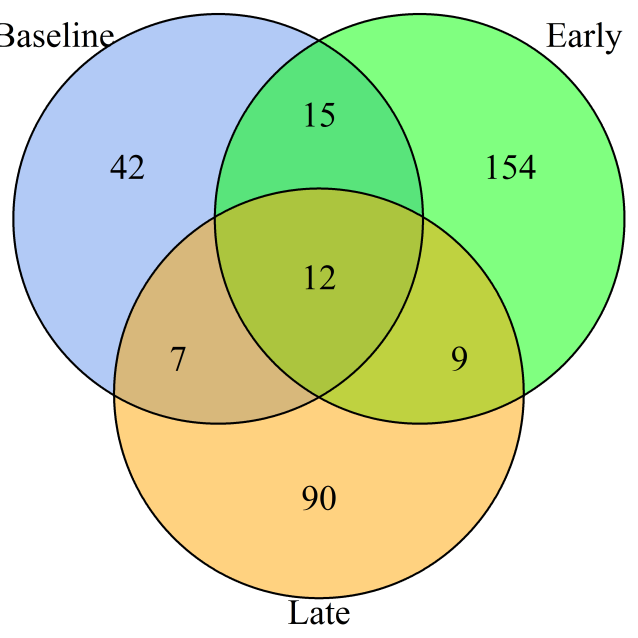


**A B C**

**Figure S1: Across-time methylation associations with expression are more stable than with humoral immune outcomes.** We show Venn-Diagrams of the number of genes shared in common between the three time points. **A)** Gene expression is highly concordant across time. This is in contrast to both **B)** HAI and **C)** B-Cell ELISPOT, for which most of the genes showing statistically significant associations with baseline methylation levels are within an individual time point. E.g. There are 90 genes for which baseline cis-methylation correlates with baseline HAI, 67 of which do not show associations with HAI at later time points. Each humoral immune outcome (HAI and B-cell) shows a small core set of genes that are associated across time points, but a diversity of genes within each time point. Baseline = Day 0; Early = Day 3; Late = Day 28.
