# Supplementary material for: System-Wide Associations between DNA-Methylation, Gene Expression, and Humoral Immune Response to Influenza Vaccination
Source: PLoS One. 2016 Mar 31;11(3):e0152034. doi: 10.1371/journal.pone.0152034 (PMC4816338; doi:10.1371/journal.pone.0152034)
Supplement: S2 Fig — (DOCX) [file pone.0152034.s002.docx]

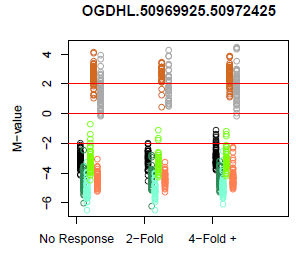

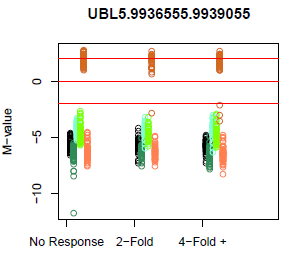

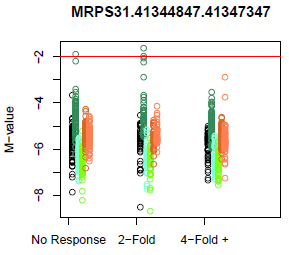


**A B C**

**Figure S2: Examples of gene promoters exhibiting different levels of consistency between CpGs sites.** Note different ordinate bounds for each plot. The title of each panel shows the gene name for the promoter sites considered, followed by the genomic bounds used. Subjects are split into three groups by HAI titer. All of the assayed CpG probes in the region are shown, each with a different color. Red horizontal lines mark -2, 0, and +2, corresponding to methylation Beta-values of 0.2, 0.5, and 0.8, respectively. Three examples are shown: **A**) Promoter with 7 CpGs, five of which are unmethylated, and two of which are generally methylated. **B**) Promoter with 6 CpGs, one of which is methylated. **C**) Promoter with 5 CpGs, all of which are unmethylated.
