# Supplementary material for: System-Wide Associations between DNA-Methylation, Gene Expression, and Humoral Immune Response to Influenza Vaccination
Source: PLoS One. 2016 Mar 31;11(3):e0152034. doi: 10.1371/journal.pone.0152034 (PMC4816338; doi:10.1371/journal.pone.0152034)
Supplement: S3 Fig — (DOCX) [file pone.0152034.s003.docx]

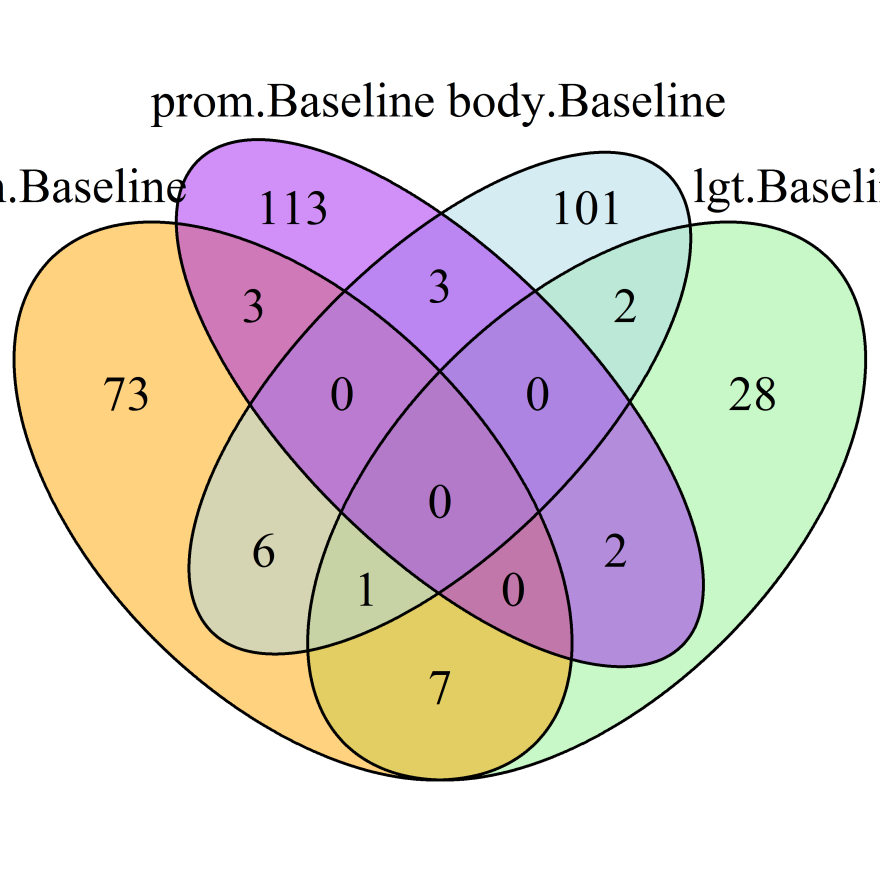

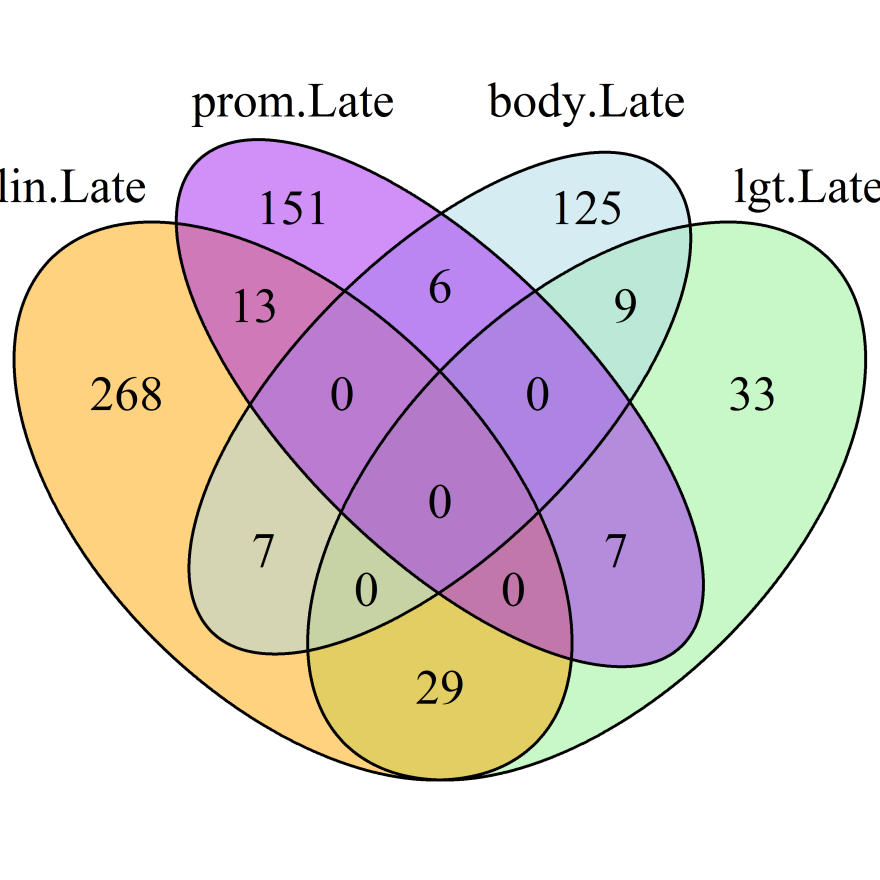


**Per-CpG**

**Linear**

**Promoter**

**Averaged**

**Body**

**Averaged**

**Per-CpG**

**Logistic**

**Per-CpG**

**Linear**

**Promoter**

**Averaged**

**Body**

**Averaged**

**Per-CpG**

**Logistic**

Baseline Late

**Figure S3: Comparison of the number of genes shared by HAI association methods.** Genes are considered from the per-CpG analyses if any cis-acting (within promoter or body) CpG has a statistically significant association with HAI. The choice of analysis type implicates largely disjoint groups of genes. Utilizing average methylation across genomic regions shows lower overlap than modeling per-CpG values, indicating large differences due to averaging. Day 0 methylation levels were used in all analyses. Baseline, Day 0 HAI titer; Late, Day 28 HAI titer.

Considering all of our analyses, there are 8 genes (0.6%) prioritized in at least 3 analyses (across baseline, early, and late HAI), 113 in at least 2 (8.9%), and 1157 in only 1 (91.1%). The 8 genes are: HLA-DQB2, RWDD2B, PTPRN2, DNAH2, HCP5, FAM24B-CUZD1, FAM24B, LOC399815.

Genes identified by per-CpG analyses are shown in bold-face type.

The 27 genes prioritized by ≥ 2 Baseline HAI comparisons are: AGBL2, ANKRD36, ATP6AP1L, BMP5, FLT4, GCNT2, **HDAC4**, **HLA-B**, **HLA-DQB2**, HMGB4, HYAL1, **IL12RB2**, IL22, MFF, **MUCL1**, **MYLIP**, NDUFS3, NPL, **PAX9**, PLEKHG5, SDHC, TBC1D12, TNXB, TOX, UNC93A, UTP23, **ZNRD1-AS1**.

The 76 genes prioritized by ≥ 2 Late HAI comparisons are: AMBP, AMMECR1L, **AP4B1-AS1**, **C11orf48**, **C11orf83**, **C2**, **C6orf10**, **C6orf147**, **C6orf52**, CCDC151, **CHST4**, DDX6, DMAP1, EIF2AK2, ETNK2, FAHD1, FASLG, FAT4, FBLL1, FIGN, GLIPR2, GPR160, HAGH, HAPLN1, **HCP5**, **HDAC4**, **HLA-DQB2**, **HMGB2**, HSF4, IFNLR1, IFRD1, KCNG4, KCNK3, **KHDC1**, **KIAA0430**, KXD1, LBX2-AS1, LINC00085, LMF1, LOC338758, **MIR484**, **MTHFD1**, NBL1, **NDE1**, OAZ3, **PAK1IP1**, **PAX7**, PCDHAC2, PCSK1, PHF17, PI16, **PITX2**, **PRMT8**, **PTPN22**, **RGR**, **RNF216**, **RNF216-IT1**, **SERTM1**, SLC7A8, SPG20, SPOCD1, STK3, TFG, TMEM254, **TPTEP1**, TSPAN5, TTPAL, UGP2, VAV3-AS1, VGLL2, **ZBTB12**, ZBTB47, ZBTB9, ZIM2, **ZNF160**, ZNF790.
