# Supplementary material for: System-Wide Associations between DNA-Methylation, Gene Expression, and Humoral Immune Response to Influenza Vaccination
Source: PLoS One. 2016 Mar 31;11(3):e0152034. doi: 10.1371/journal.pone.0152034 (PMC4816338; doi:10.1371/journal.pone.0152034)
Supplement: S4 Fig — (DOCX) [file pone.0152034.s004.docx]

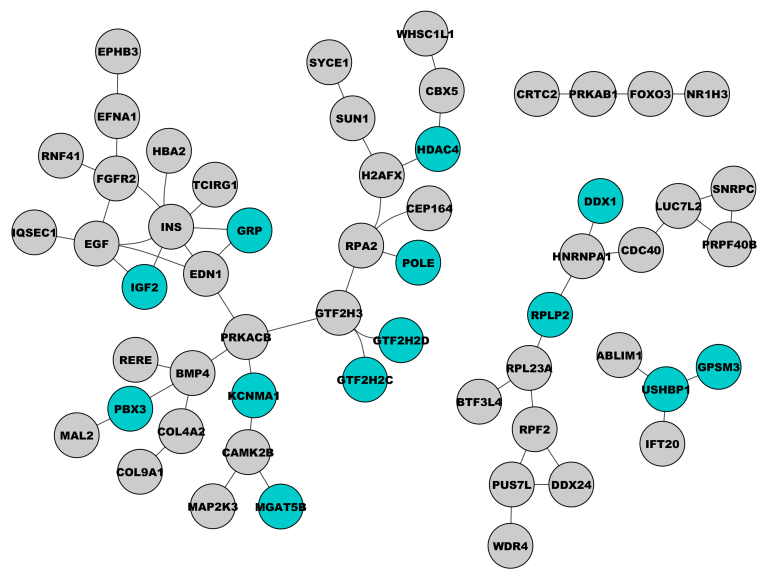

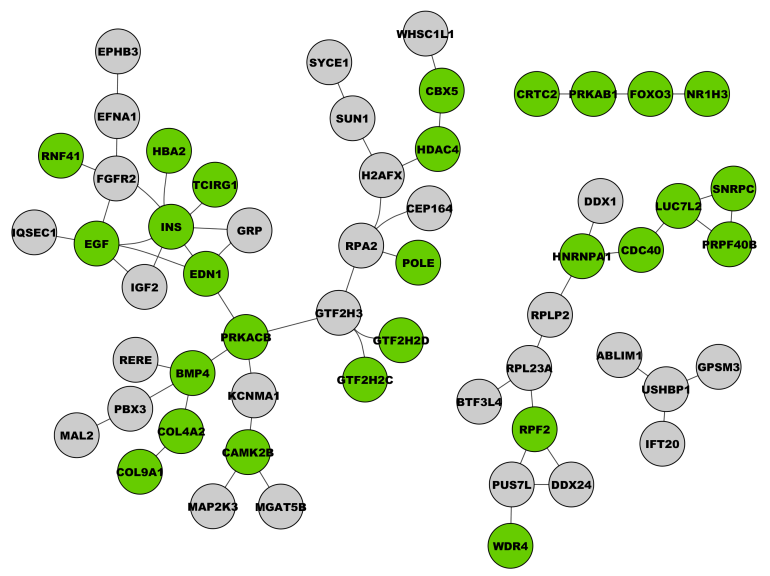

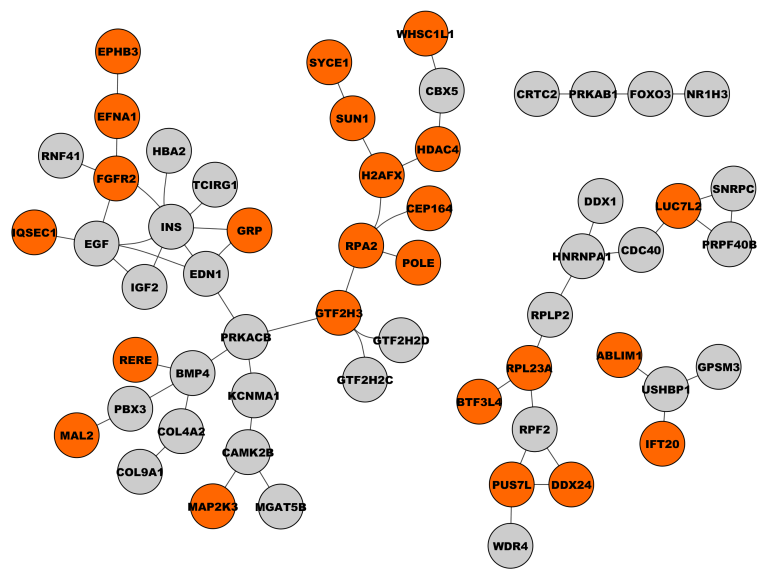

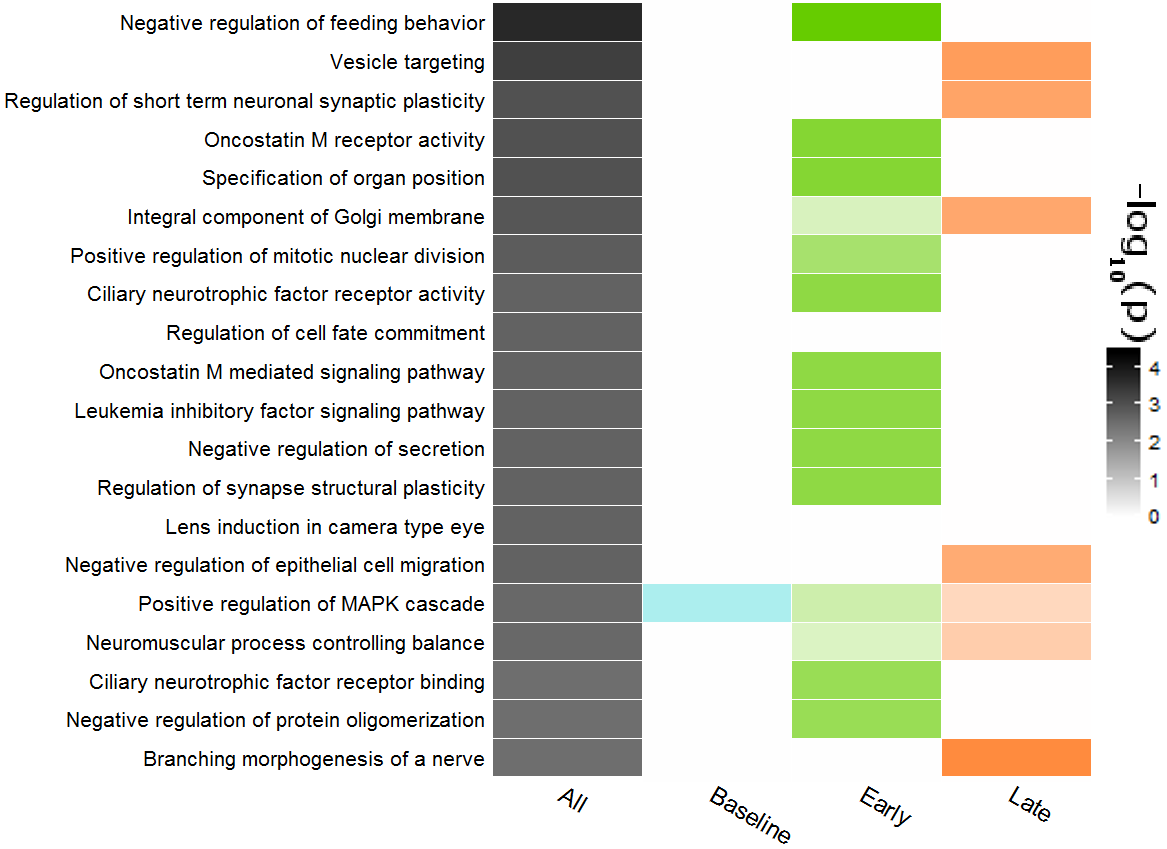


**A B**

**C**

**Figure S4: Examples of regulatory network interactions implicated by CpG sites whose methylation levels correlate with B-cell ELISPOT response.** Color indicates at which time the CpG is associated with a change in HAI titer. Such interactions indicate how multiple methylation marks can affect related biologic functions and how their combination makes a more unified picture between genes that initially distinguish baseline response (Day 0) and the magnitude of response (change from Day 0 to Day28).
