# Supplementary material for: System-Wide Associations between DNA-Methylation, Gene Expression, and Humoral Immune Response to Influenza Vaccination
Source: PLoS One. 2016 Mar 31;11(3):e0152034. doi: 10.1371/journal.pone.0152034 (PMC4816338; doi:10.1371/journal.pone.0152034)
Supplement: S5 Fig — (DOCX) [file pone.0152034.s005.docx]

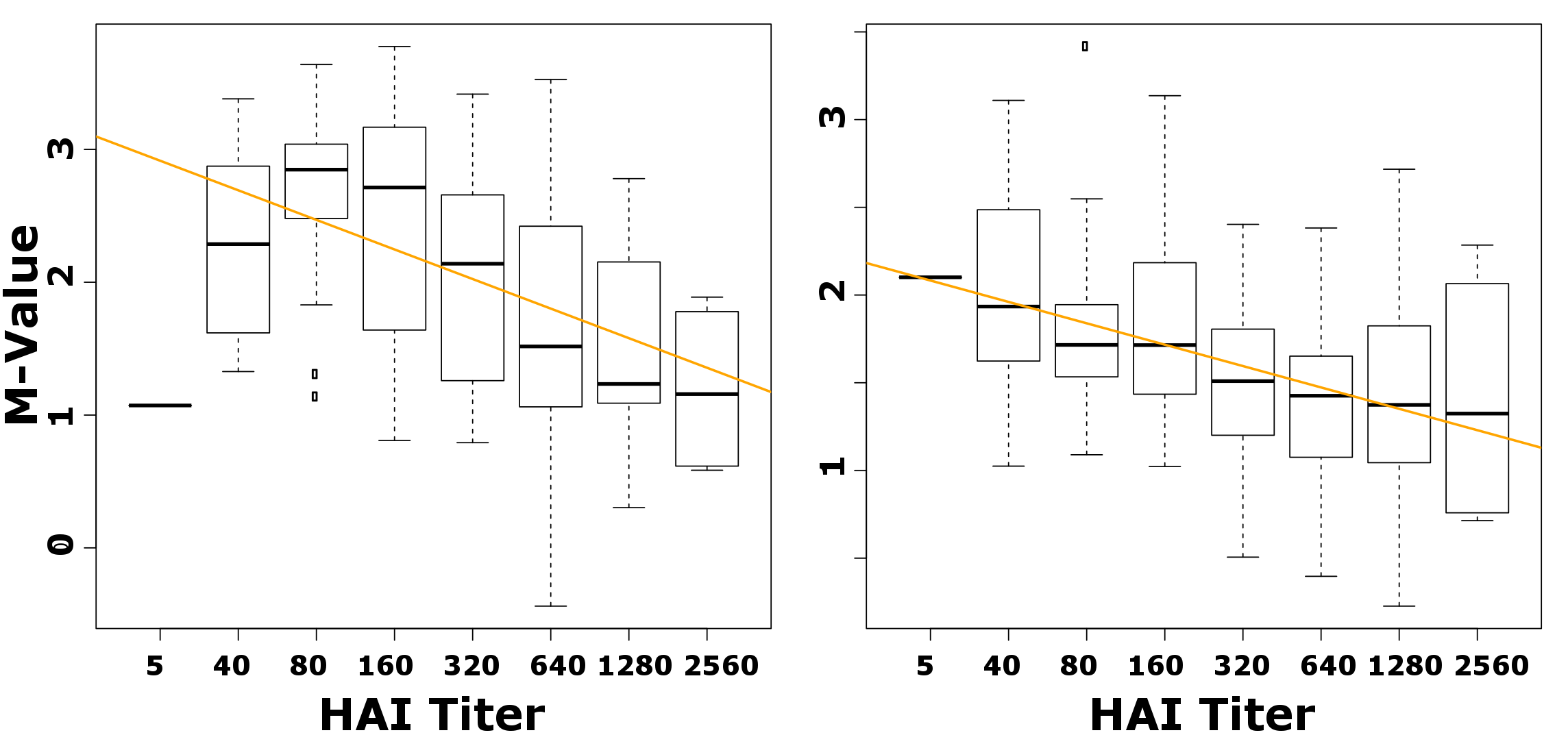


**A B**

**Figure S5: The most significant associations from linear models of methylation versus HAI titer are for A) HLA-DQB2 and B) HLA-B**. HAI titer is expressed by dilution coefficient, where, for example, “40” indicates 1:40 dilution. The M-value observed for patients with each level of HAI titer are summarized as a boxplot where a thick line marks the median, a box surrounds the interquartile range (middle 50% of data points), and dashed lines extend to the full data range. The linear model fit is shown as an orange line. See Table 2 for further results.
