# Supplementary material for: System-Wide Associations between DNA-Methylation, Gene Expression, and Humoral Immune Response to Influenza Vaccination
Source: PLoS One. 2016 Mar 31;11(3):e0152034. doi: 10.1371/journal.pone.0152034 (PMC4816338; doi:10.1371/journal.pone.0152034)
Supplement: S6 Fig — (DOCX) [file pone.0152034.s006.docx]

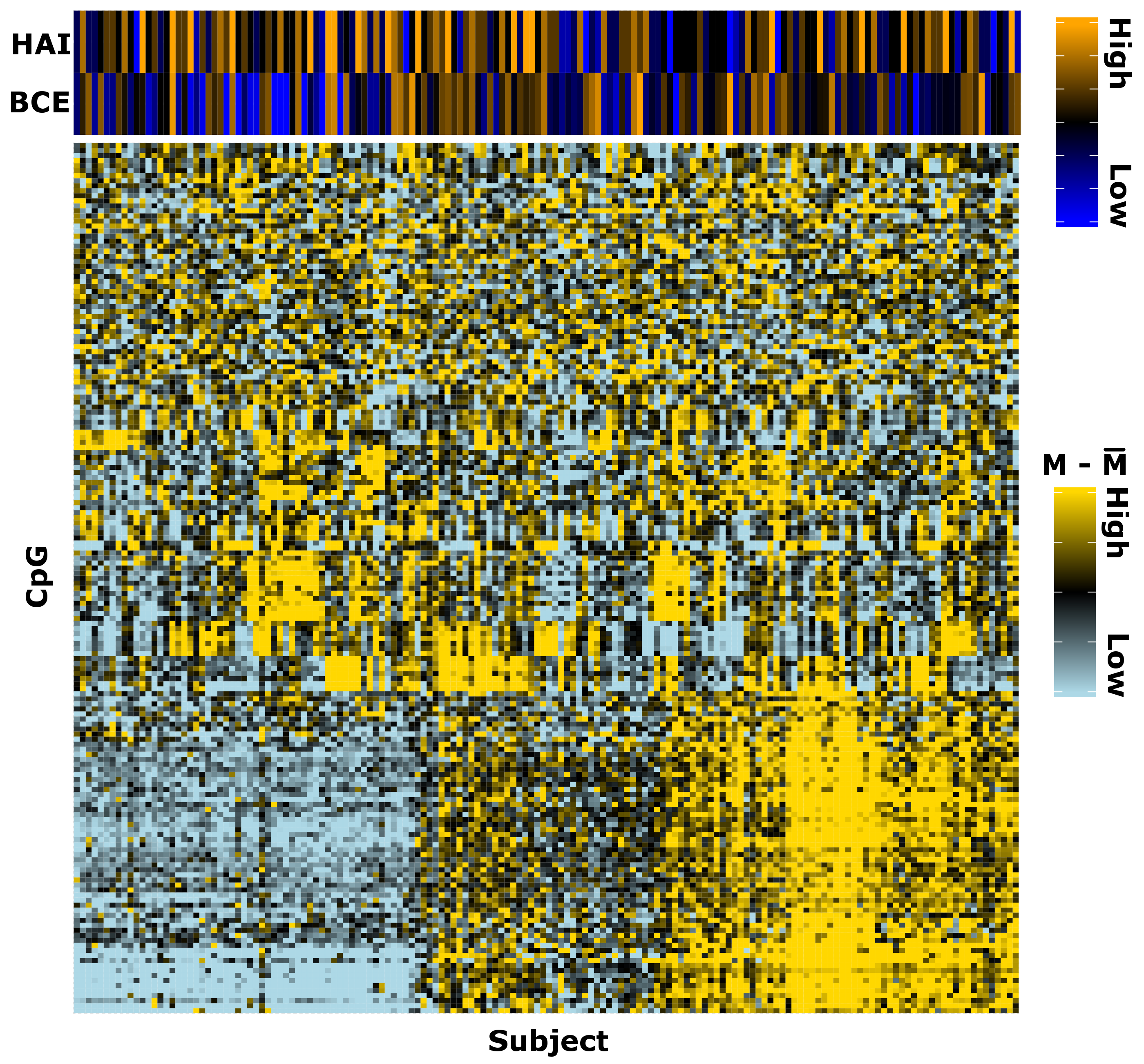


**Figure S5: Unsupervised clustering reveals moderate concordance between baseline (pre-vaccination) B-cell ELISPOT (BCE) levels and CpG methylation.** The CpG heatmap shows the median-centered level for each probe at baseline. Probes shown are the top quartile (by variance, n = 173) of CpGs with a statistical association with late response (day 28 HAI level). Participants with low methylation within a particular cluster of CpGs (red box) show lower baseline log_2_ B-cell ELISPOT values (2.7±1.8) than participants with high methylation of levels (blue box) of the same CpGs (3.4±1.4).
