# Supplementary material for: System-Wide Associations between DNA-Methylation, Gene Expression, and Humoral Immune Response to Influenza Vaccination
Source: PLoS One. 2016 Mar 31;11(3):e0152034. doi: 10.1371/journal.pone.0152034 (PMC4816338; doi:10.1371/journal.pone.0152034)
Supplement: S1 Table — (DOCX) [file pone.0152034.s007.docx]

**Table S1: Most frequent TFs binding to the most informative CpGs.**

|  |  | Top 10 TFs Binding at trans-CpGs (of 3,057) | | | | | | | | | |
| --- | --- | --- | --- | --- | --- | --- | --- | --- | --- | --- | --- |
| Top 10 | TF | POLR2A | CTCF | MAX | MYC | EZH2 | RAD21 | EP300 | YY1 | TBP | MAZ |
|  | # CpGs | 547 | 435 | 348 | 317 | 298 | 265 | 264 | 258 | 227 | 223 |
|  |  | trans-CpGs in HAI linear model results with p < 0.001 (of 154)  (Top-10 most common, followed by any others with significant enrichment) | | | | | | | | | |
| Top 10 | TF | POLR2A | CTCF | EZH2 | MYC | MAX | RAD21 | YY1 | TBP | CEBPB | SMC3 |
|  | # CpGs | 30 | 29 | 21 | 19 | 17 | 16 | 14 | 14 | 13 | 12 |
|  | P-Value | 4.87E-01 | 5.46E-01 | **4.79E-03** | 5.85E-01 | 7.05E-01 | 5.52E-01 | 5.24E-01 | 2.69E-01 | 6.96E-01 | 2.25E-01 |
|  | OR | 0.8 | 1.2 | 3.6 | 1.2 | 1.3 | 1.3 | 1.4 | 1.8 | 0.8 | 1.9 |
| P < 0.1 #CpG ≥ 5 | TF | CTBP2 | EP300 | SUZ12 |  | | | | | | |
|  | # CpGs | 11 | 10 | 7 |  |  |  |  |  |  |  |
|  | P-Value | **2.34E-02** | **8.37E-02** | **7.33E-02** |  |  |  |  |  |  |  |
|  | OR | 6.7 | 0.5 | 5.5 |  |  |  |  |  |  |  |
|  |  | trans-CpGs with B-cell ELISPOT linear model results with p < 0.001 (of 135)  (Top-10 most common, followed by any others with significant enrichment) | | | | | | | | | |
| Top 10 | TF | YY1 | POLR2A | MAX | JUND | MYC | CEBPB | TBP | REST | CTCF | TCF7L2 |
|  | # CpGs | 22 | 20 | 19 | 15 | 14 | 14 | 13 | 13 | 13 | 12 |
|  | P-Value | **2.20E-02** | **8.96E-02** | 2.52E-01 | 5.36E-01 | 1.00E+00 | 1.00E+00 | 2.45E-01 | 2.45E-01 | 1.99E-01 | 4.86E-01 |
|  | OR | 2.5 | 0.6 | 1.6 | 1.3 | 1 | 1 | 2 | 1.8 | 0.6 | 1.4 |
| P < 0.1  #CpG ≥ 5 | TF | FOXP2 |  | | | | | | | | |
|  | # CpGs | 10 |  |  |  |  |  |  |  |  |  |
|  | P-Value | **8.81E-02** |  |  |  |  |  |  |  |  |  |
|  | OR | 3.3 |  |  |  |  |  |  |  |  |  |

We count the number of CpG sites with a known Transcription Factor binding sites (TFBSs) overlapping enhancer-associated trans-CpGs with significant immune outcome associations. We report the ten most frequently observed TFBSs, as well as any less frequent that are significantly different from the expected prevalence across the genome.
