# Supplementary material for: System-Wide Associations between DNA-Methylation, Gene Expression, and Humoral Immune Response to Influenza Vaccination
Source: PLoS One. 2016 Mar 31;11(3):e0152034. doi: 10.1371/journal.pone.0152034 (PMC4816338; doi:10.1371/journal.pone.0152034)
Supplement: S2 Table — (DOCX) [file pone.0152034.s008.docx]

**Table S2 : Influenza HAI logistic models utilizing Day 0 methylation**.

| CpG | OR^†^ | P-Value | Q-value | GenomicRegion | Gene |
| --- | --- | --- | --- | --- | --- |
| cg03249171 | 3.36 | 8.00E-05 | 7.82e-01 | - | - |
| cg07869256 | 3.47 | 9.18E-05 | 7.82e-01 | Promoter | MUCL1 |
| cg02914652 | 3.06 | 1.11E-04 | 7.82e-01 | Open, TF | - |
| cg23923934 | 0.32 | 1.25E-04 | 7.82e-01 | GeneBody | HLA-B |
| cg06480496 | 2.62 | 1.74E-04 | 7.82e-01 | Promoter | HCP5 |
| cg01168339 | 2.83 | 1.77E-04 | 7.82e-01 | Promoter;GeneBody | LOC100506668;DYNLL1 |
| cg13555933 | 2.42 | 2.63E-04 | 7.82e-01 | - | - |
| cg27580026 | 0.41 | 2.74E-04 | 7.82e-01 | Promoter | FNDC4;GCKR |
| cg01858895 | 0.37 | 2.82E-04 | 7.82e-01 | Open, TF | - |
| cg03556243 | 2.53 | 3.14E-04 | 7.82e-01 | Promoter;GeneBody | ZBTB20 |
| cg23223756 | 0.39 | 3.17E-04 | 7.82e-01 | Promoter | ZNRD1-AS1;ZNRD1 |
| cg10594709 | 0.41 | 3.18E-04 | 7.82e-01 | Open, TF | - |
| cg11194925 | 2.75 | 3.52E-04 | 7.82e-01 | GeneBody | PAX9 |
| cg24211400 | 0.45 | 3.56E-04 | 7.82e-01 | Promoter | CITED2 |
| cg12807946 | 0.43 | 4.13E-04 | 7.82e-01 | Open, No TF | - |
| cg15404791 | 2.33 | 4.21E-04 | 7.82e-01 | GeneBody | MYLIP |
| cg07703701 | 2.46 | 4.31E-04 | 7.82e-01 | GeneBody | PDE4DIP |
| cg18043273 | 0.45 | 5.17E-04 | 7.82e-01 | GeneBody | SLC6A1 |
| cg26964544 | 0.40 | 5.38E-04 | 7.82e-01 | GeneBody | THSD4 |
| cg07992625 | 0.40 | 5.39E-04 | 7.82e-01 | Promoter | ATF7 |

^†^We express the effect size of logistic regression models in terms of an odds ratio (OR) for the odds of being a responder if the participant is at Q3 (the 75^th^ percentile) of methylation (per site), versus a participant at Q1 (the 25^th^ percentile).
