# Supplementary material for: System-Wide Associations between DNA-Methylation, Gene Expression, and Humoral Immune Response to Influenza Vaccination
Source: PLoS One. 2016 Mar 31;11(3):e0152034. doi: 10.1371/journal.pone.0152034 (PMC4816338; doi:10.1371/journal.pone.0152034)
Supplement: S3 Table — (DOCX) [file pone.0152034.s009.docx]

**Table S3: Spearman correlation between the average baseline methylation level (across probes) of gene promoters and gene bodies with influenza HAI.**

| Gene Promoter | r | p-value | q-value | Gene Body | r | p-value | q-value |
| --- | --- | --- | --- | --- | --- | --- | --- |
| MIEN1 | 0.29 | 2.62E-4 | 9.99E-1 | LOC550112 | 0.29 | 2.35E-4 | 9.99E-1 |
| ATP6AP1L | -0.27 | 5.48E-4 | 9.99E-1 | BTRC | 0.28 | 2.93E-4 | 9.99E-1 |
| ZNF330 | 0.27 | 6.80E-4 | 9.99E-1 | ATXN10 | 0.28 | 3.18E-4 | 9.99E-1 |
| HSD17B7 | 0.26 | 7.93E-4 | 9.99E-1 | MSX1 | 0.28 | 3.71E-4 | 9.99E-1 |
| GCNT2 | 0.26 | 8.27E-4 | 9.99E-1 | E2F7 | 0.27 | 4.82E-4 | 9.99E-1 |
| HSD17B7P2 | 0.26 | 8.27E-4 | 9.99E-1 | HERC2 | 0.27 | 6.39E-4 | 9.99E-1 |
| ZBTB20-AS1 | 0.26 | 8.75E-4 | 9.99E-1 | SLC9C1 | 0.27 | 7.57E-4 | 9.99E-1 |
| IL22 | 0.26 | 9.30E-4 | 9.99E-1 | MGC2752 | 0.26 | 8.18E-4 | 9.99E-1 |
| XKR5 | -0.26 | 1.15E-3 | 9.99E-1 | AGBL2 | 0.26 | 8.26E-4 | 9.99E-1 |
| LOC100652791 | -0.26 | 1.15E-3 | 9.99E-1 | GCNT2 | 0.26 | 8.27E-4 | 9.99E-1 |
| ARHGDIB | -0.25 | 1.43E-3 | 9.99E-1 | NANP | 0.26 | 1.01E-3 | 9.99E-1 |
| NPL | -0.25 | 1.46E-3 | 9.99E-1 | C6orf7 | 0.25 | 1.22E-3 | 9.99E-1 |
| OLFML2B | 0.25 | 1.52E-3 | 9.99E-1 | PGM3 | 0.25 | 1.26E-3 | 9.99E-1 |
| BMP5 | 0.25 | 1.61E-3 | 9.99E-1 | FLT4 | 0.25 | 1.27E-3 | 9.99E-1 |
| ATP5EP2 | 0.25 | 1.62E-3 | 9.99E-1 | SHE | 0.25 | 1.31E-3 | 9.99E-1 |
| GYS2 | 0.25 | 1.66E-3 | 9.99E-1 | ATP2A3 | 0.25 | 1.35E-3 | 9.99E-1 |
| GNAS-AS1 | -0.25 | 1.78E-3 | 9.99E-1 | GAS2 | -0.25 | 1.39E-3 | 9.99E-1 |
| GNAS | -0.24 | 1.93E-3 | 9.99E-1 | CYSTM1 | -0.25 | 1.59E-3 | 9.99E-1 |
| RFWD2 | 0.24 | 2.01E-3 | 9.99E-1 | TMX2-CTNND1 | 0.25 | 1.82E-3 | 9.99E-1 |
| NEU3 | 0.24 | 2.13E-3 | 9.99E-1 | BAMBI | -0.24 | 1.95E-3 | 9.99E-1 |
