# Supplementary material for: System-Wide Associations between DNA-Methylation, Gene Expression, and Humoral Immune Response to Influenza Vaccination
Source: PLoS One. 2016 Mar 31;11(3):e0152034. doi: 10.1371/journal.pone.0152034 (PMC4816338; doi:10.1371/journal.pone.0152034)
Supplement: S4 Table — (DOCX) [file pone.0152034.s010.docx]

**Table S4: B-cell ELISPOT response linear regression models.**

| CpG ($M_{i}$) | ∆B-cell for ∆*M_i_* _Q3 to Q1_^†^ | p-value | q-value | GenomicRegion | Gene |
| --- | --- | --- | --- | --- | --- |
| cg00816037 | -0.62 | 1.34E-6 | 0.14 | GeneBody | PIEZO1 |
| cg10369242 | -0.55 | 3.71E-6 | 0.15 | Promoter | KRT7 |
| cg08487581 | 0.37 | 4.53E-6 | 0.15 | Open, No TF | - |
| cg05165436 | 0.50 | 6.71E-6 | 0.17 | Open, TF | - |
| cg26399903 | -0.56 | 1.14E-5 | 0.23 | - | - |
| cg13944037 | 0.21 | 2.43E-5 | 0.41 | Promoter | GUCA1B |
| cg05114739 | -0.47 | 4.44E-5 | 0.64 | GeneBody | HDAC4 |
| cg12587766 | 0.37 | 7.10E-5 | 0.79 | Promoter;GeneBody | LIFR |
| cg00144039 | 0.42 | 7.60E-5 | 0.79 | GeneBody | ERICH1-AS1 |
| cg26832639 | 0.37 | 7.81E-5 | 0.79 | Promoter | KLK5 |
| cg11370814 | -0.46 | 1.04E-4 | 0.79 | Open, TF | - |
| cg02992546 | 0.47 | 1.05E-4 | 0.79 | Promoter | ANKRD30B |
| cg25802888 | 0.53 | 1.06E-4 | 0.79 | Promoter | AURKC |
| cg16385533 | 0.46 | 1.16E-4 | 0.79 | Open, No TF | - |
| cg00276799 | -0.40 | 1.17E-4 | 0.79 | GeneBody | ELMOD1 |
| cg14148156 | 0.42 | 1.52E-4 | 0.81 | GeneBody | TMEM8B |
| cg19975849 | -0.35 | 1.53E-4 | 0.81 | Open, TF | - |
| cg24037270 | 0.44 | 1.53E-4 | 0.81 | GeneBody | SGMS1 |
| cg17123334 | -0.35 | 1.59E-4 | 0.81 | GeneBody | LOC100507266 |
| cg13325489 | -0.42 | 1.60E-4 | 0.81 | - | - |

Log2 of (median across replicates) stimulated B-cell ELISPOT values was used as response. All models were adjusted by baseline B-cell ELISPOT values.

^†^ We express the effect size of linear regression models in terms of the change in B-cell ELISPOT predicted (from the model) for participants at the Q3 (75^th^) percentile of methylation M-value relative to Q1 (25^th^).
