# Supplementary material for: System-Wide Associations between DNA-Methylation, Gene Expression, and Humoral Immune Response to Influenza Vaccination
Source: PLoS One. 2016 Mar 31;11(3):e0152034. doi: 10.1371/journal.pone.0152034 (PMC4816338; doi:10.1371/journal.pone.0152034)
Supplement: S5 Table — (DOCX) [file pone.0152034.s011.docx]

**Table S5: Spearman correlation between the average methylation level (across probes) of gene promoters and gene bodies with the change in B-cell ELISPOT from Day 0 to Day 28.**

| Gene Promoter | r^†^ | p-value | q-value | Gene Body | r | p-value | q-value |
| --- | --- | --- | --- | --- | --- | --- | --- |
| TTC5 | 0.37 | 2.37E-6 | 4.25e-2 | MS4A4A | 0.28 | 3.12E-4 | 9.99e-1 |
| FRS2 | -0.30 | 1.01E-4 | 8.99e-1 | NEDD4 | -0.28 | 3.55E-4 | 9.99e-1 |
| EFNA4 | -0.29 | 1.89E-4 | 9.99e-1 | FKBP14 | 0.28 | 3.75E-4 | 9.99e-1 |
| SELRC1 | -0.28 | 3.21E-4 | 9.99e-1 | CREM | -0.28 | 4.50E-4 | 9.99e-1 |
| NBL1 | 0.27 | 5.04E-4 | 9.99e-1 | ERVFRD-1 | -0.27 | 5.51E-4 | 9.99e-1 |
| KIAA1279 | -0.27 | 5.75E-4 | 9.99e-1 | SLC32A1 | -0.27 | 7.22E-4 | 9.99e-1 |
| RUNX3 | 0.27 | 5.76E-4 | 9.99e-1 | SOX9 | -0.26 | 8.56E-4 | 9.99e-1 |
| PCDHA6 | 0.27 | 5.83E-4 | 9.99e-1 | ACBD4 | -0.26 | 9.79E-4 | 9.99e-1 |
| TFF1 | 0.27 | 5.88E-4 | 9.99e-1 | MOB3B | 0.25 | 1.25E-3 | 9.99e-1 |
| CBY3 | 0.27 | 6.14E-4 | 9.99e-1 | NCMAP | 0.25 | 1.34E-3 | 9.99e-1 |
| DEFA5 | 0.27 | 7.02E-4 | 9.99e-1 | ZNF804A | -0.25 | 1.51E-3 | 9.99e-1 |
| HS2ST1 | 0.26 | 7.87E-4 | 9.99e-1 | DLG1-AS1 | -0.25 | 1.54E-3 | 9.99e-1 |
| LYG1 | 0.26 | 8.01E-4 | 9.99e-1 | HCRTR1 | -0.25 | 1.60E-3 | 9.99e-1 |
| E2F6 | -0.26 | 9.29E-4 | 9.99e-1 | KIF18A | 0.25 | 1.78E-3 | 9.99e-1 |
| WDR6 | -0.26 | 1.09E-3 | 9.99e-1 | GLYAT | 0.24 | 2.01E-3 | 9.99e-1 |
| CELF5 | 0.26 | 1.12E-3 | 9.99e-1 | PTGR2 | -0.24 | 2.11E-3 | 9.99e-1 |
| LOC643542 | 0.26 | 1.15E-3 | 9.99e-1 | TMEM8B | -0.24 | 2.11E-3 | 9.99e-1 |
| AXIN2 | -0.25 | 1.30E-3 | 9.99e-1 | GPR4 | -0.24 | 2.11E-3 | 9.99e-1 |
| RPP25 | 0.25 | 1.31E-3 | 9.99e-1 | POM121C | -0.24 | 2.24E-3 | 9.99e-1 |
| YWHAZ | -0.25 | 1.32E-3 | 9.99e-1 | C14orf178 | -0.24 | 2.52E-3 | 9.99e-1 |

^†^r is the Spearman’s correlation coefficient.
